# Clinical Efficacy of Tumor Antigen-Pulsed DC Treatment for High-Grade Glioma Patients: Evidence from a Meta-Analysis

**DOI:** 10.1371/journal.pone.0107173

**Published:** 2014-09-12

**Authors:** Jun-Xia Cao, Xiao-Yan Zhang, Jin-Long Liu, Duo Li, Jun-Li Li, Yi-Shan Liu, Min Wang, Bei-Lei Xu, Hai-Bo Wang, Zheng-Xu Wang

**Affiliations:** 1 Biotherapy Center, the General Hospital of Beijing Military Command, Beijing, People's Republic of China; 2 Tsinghua-Peking Center for Life Sciences, Laboratory of Dynamic Immunobiology, School of Medicine, School of Life Sciences, Tsinghua University, Beijing, People's Republic of China; Cedars-Sinai Medical Center, United States of America

## Abstract

**Background:**

The effectiveness of immunotherapy for high-grade glioma (HGG) patients remains controversial. To evaluate the therapeutic efficacy of dendritic cells (DCs) alone in the treatment of HGG, we performed a systematic review and meta-analysis in terms of patient survival with relevant published clinical studies.

**Materials and methods:**

A total of 409 patients, including historical cohorts, nonrandomized and randomized controls with HGG, were selected for the meta-analysis.

**Results:**

The treatment of HGG with DCs was associated with a significantly improved one-year survival (OS) (*p*<0.001) and 1.5-, 2-, 3-, 4-, and 5-year OS (*p*<0.001) compared with the non-DC group. A meta-analysis of the patient outcome data revealed that DC immunotherapy has a significant influence on progression-free survival (PFS) in HGG patients, who showed significantly improved 1-,1.5-, 2-, 3- and 4-year PFS (*p*<0.001). The analysis of Karnofsky performance status (KPS) demonstrated no favorable results for DC cell therapy arm (*p* = 0.23).The percentages of CD3^+^CD8^+^ and CD3^+^CD4^+^ T cells and CD16^+^ lymphocyte subset were not significantly increased in the DC group compared with the baseline levels observed before treatment (*p*>0.05), whereas CD56^+^ lymphocyte subset were significantly increased after DC treatment (*p* = 0.0001). Furthermore, the levels of IFN-γ in the peripheral blood of HGG patients, which reflect the immune function of the patients, were significantly increased after DC immunotherapy (*p*<0.001).

**Conclusions:**

Thus, our meta-analysis showed that DC immunotherapy markedly prolongs survival rates and progression-free time, enhances immune function, and improves the efficacy of the treatment of HGG patients.

## Introduction

High-grade gliomas (HGGs) have an incidence currently estimated at 14,000 new diagnoses per year, according to the 2007 World Health Organization (WHO) classification, which includes patients with anaplastic astrocytomas (WHO grade III) and with glioblastoma multiforme (GBM, WHO grade IV) [1]. GBM is the most common and most malignant glioma in adults and represents approximately 75% of all newly diagnosed glioma cases; moreover, the prognosis of these patients remains poor, with a median survival of less than 15 months, despite the use of trimodal therapy [2]. Indeed, there is no conventional treatment that specifically targets tumor cells and spares normal brain parenchyma. Immunologic approaches are theoretically able to trace, identify, and kill dispersed tumor cells with great accuracy and are being tested to enhance the response of these tumors to existing therapy and/or to stimulate innate immune responses [3].

Based on previous studies, it was assumed that immune reactions do not occur in the brain because of the blood-brain barrier (BBB); however, we now have an in-depth understanding that the central nervous system maintains a two-way communication network with the immune system, with each having a profound influence on the other [4]. Several studies have clarified that lymphocytes and antigen-presenting cells (APCs), including macrophages and dendritic cells (DCs), are able to cross the blood-brain barrier and migrate to a tumor within the brain parenchyma [5]. Thus, in phase I and phase II trials, adoptive immunotherapy including lymphokine-activated killer cells (LAK), cytotoxic T lymphocytes (CTLs) and tumor-infiltrating T lymphocytes (TILs) and active immunotherapy using autologous tumor cells (ATCs) and DCs have demonstrated clinical efficacy, suggesting that immunotherapy may be a useful strategy to combat HGGs [6].

DCs are the most potent APCs in the human body. Importantly, DCs can cross the BBB and traffic into perivascular and parenchymal spaces in the glioma [5], [7]. An important milestone has been reached with the recent approval in 2010 of sipuleucel-T (Provenge), the first DC vaccine for hormone-resistant metastatic prostate cancer. This vaccine is primarily an active immunologic agent with proven activity against solid tumors [8]. In HGG, a cohort comparison trial involving 45 children, HGG-IMMUNO-2003, has been conducted since 2001 to implement and improve immunotherapeutic approaches [9]. Additionally, another clinical trial of 77 newly diagnosed glioblastoma patients was performed [10]. In addition, a Phase I/II single-arm clinical trial, HGG-2006, was designed and conducted using 117 patients [11]. In brief, all of the data showed a remarkable overall survival (OS) compared with the generally expected progression of this disease. Thus, both hope and challenges exist for DC-based immunotherapy. These data compelled the design of the current prospective placebo-controlled, double-blind Phase IIb stratified randomized clinical trial (EudraCT number 2009-018228-14) and the Phase III study of DCVax in GBM, which has been registered at ClinicalTrials.gov (NCT00045968).

Unfortunately, due to profound tumor-associated mechanisms of immunosuppression and evasion, immunotherapeutic strategies have thus far not translated into clinical success [12]. There are several reviews that summarize more than 21 DC clinical trials that were performed in HGG in which up to 500 patients were involved, excluding controls. These studies always used historical or nonrandomized cohorts due to the disease's malignancy [13]–[15]. However, evidence from the meta-analysis through logistic regression regarding the OS, PFS, and other outcomes of the therapy remains scarce. Here, we addressed the effect of the autologous tumor antigen-pulsed DCs on the treatment of glioma patients in terms of survival compared with historical cohorts or nonrandomized and randomized control groups.

## Materials and Methods

### 2.1 Literature search and inclusion and exclusion criteria

The trials analyzed in this study were identified through an electronic search of the PubMed database, the Cochrane Central Registry of Controlled Trials, the Wanfang Database, the China Science and Technology Periodical Database, China Journal Net, reference lists of published trials, and relevant review articles. The search strategy included the medical subject headings “glioma”, “immunotherapy”, “dendritic cells”, and free text search. No language limits were applied. The initial search was performed on Nov 2013 and was updated in Jan 2014. Furthermore, manual searches were performed in reference lists and conference proceedings of the American Society of Clinical Oncology (ASCO) Annual Meetings and the European Cancer Conference (ECCO). We excluded abstracts that were never subsequently published as full papers and studies on animals and cell lines.

### 2.2 Study selection and data extraction

We collected various sets of information, including the authors' names, journal and year of publication, sample size per arm, newly or recurrent, regimen used, median or mean age of the patients, Karnofsky performance status (KPS), DC antigen, delivery route and dose, and characteristics of the study design (i.e., whether the trial reported the mode of randomization, allocation concealment, description of withdrawals per arm, and blinding) for all of the trials included in the study. The data were independently screened by two reviewers.

### 2.3 Definition of outcome measures

Overall survival (OS) was defined as the time from the initiation of treatment until death from any cause. The secondary endpoint was progression-free survival (PFS), which was documented and extracted for analysis. Quality of Life (QoL) was assessed by the KPS. The immune response was assessed by evaluating and comparing the data of surface phenotype of the patients' peripheral blood lymphocytes by FACScan from the recruited papers, including CD3^+^, CD4^+^, CD8^+^, CD16^+^ and CD56^+^ of each study. Furthermore, we approximately collected the data of CD3^+^CD8^+^ and CD3^+^CD4^+^ as the T cell subpopulation and CD16^+^ and CD56^+^ as other cell subset. In addition we also extracted the data of the cytokine IFN-γ tested by ELISA kit from the included papers.

### 2.4 Statistical analysis

The analysis was performed using Review Manager Version 5.0 (Nordic Cochran Centre, Copenhagen, Denmark). In our meta-analysis, we compared the immunotherapy-containing arms of the selected trials to the respective non-immunotherapy arms. The treatment effects are reflected by the odds ratios (OR) for OS and PFS. The OS and PFS data in each arm were extracted from each included study, and the pooled odds ratio (OR) was calculated through the Mantel and Haenszel method. A pooled OR<1 indicated a lower recurrence or lower survival in the immunotherapy arm. We used Cochran's Q test, a chi-squared test with a df equal to the number of studies minus one that tests the null hypothesis and demonstrates whether the difference among the studies based on the OR is due to chance, to evaluate whether the studies' results were homogeneous. Also calculated in the analysis was the quantity I^2^, which describes the percentage of variation across studies that is due to heterogeneity rather than chance. Generally speaking, I^2^ values of 25% represent low heterogeneity, and subsequently, I^2^ values of 50% and 75% were used as evidence of moderate and high heterogeneity, respectively. When no statistically significant heterogeneity existed, the OR was calculated with a fixed-effect model; otherwise, a random-effect model was employed. *P*-values of <0.05 were considered to be statistically significant. All reported *P*-values resulted from two-sided versions of the respective tests [16].

## Results

### 3.1 Selection of the trials

**Figure 1:**
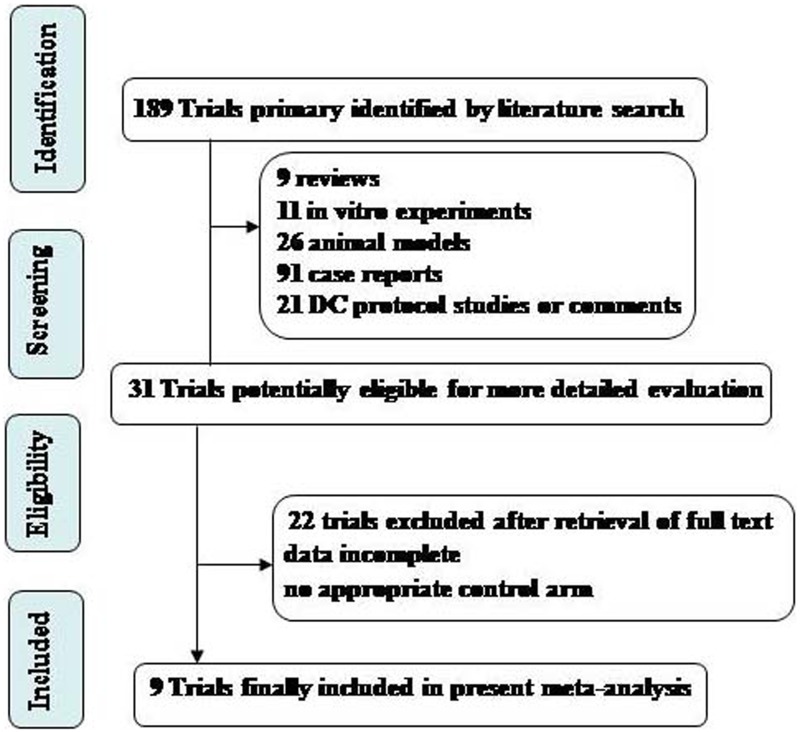
Flow diagram showing record identification, screening and study inclusion process.

The electronic search yielded 189 references. After a title and abstract review, 158 publications were excluded for different reasons (nine for being review articles, 11 for using *in vitro* experiments, 26 for being animal models, 91 for being case reports, and 21 for being DC protocol studies or comments) (Tables S1–S5 in File S1). A total of 31 clinical trials were selected as potentially relevant, and their full texts were retrieved for a more detailed assessment. We then excluded 22 of these 31 studies for not having a control arm or not providing detailed patient clinical data and details on the therapeutic response (Table S6 in File S1). The procedure used to select the clinical trials is shown in Figure 1. As a result, 9 articles reporting clinical trials of DC-based therapy were selected for the meta-analysis [17]–[25] (Table S7 in File S1).

### 3.2 Characteristics of DC cell-based therapy

After the selection process, 9 eligible trials with a total of 409 patients to date were included in the present analysis. All of the trials were fully published: three phase I trials [20], [21], [23], five phase I/II trials [17], [18], [19], [22], [24] and one phase II trials [25]. The clinical data of the trials are shown in Table 1. The median age of the included patients was <50 years. The WHO grade was mainly IV for the included HGG patients. All of the patients had experienced surgery (ST, surgical resection), chemotherapy (CT, chemical therapy), radiotherapy (RT, radiation therapy), and intra-cellular hyperthermia (ICH). The included patients were mainly recurrent containing some of new ones, and also only one trial recruited the new patients [25], which have been listed on Table 1. The patients' KPS have all been reported before immunotherapy and the value was mainly more than 60, but after the treatment only two of them reported [17], [25]. Additionally, most of the included patients received the DC therapy without any other simultaneous treatment, and the controls were four historical cohorts [17], [18], [20], [23], three nonrandomized cohorts [19], [21], [22] and two randomized cohorts [24], [25].

**Table 1:** Clinical information of the eligible trials for the meta-analysis.

Trial reference	Tumor characteristics WHO grade	Clinical trial phase	Patients (male) and control	Median age	Pre- Therapy KPS	Previous treatment	DC Arm Injection	DC regimens	Culture of DC cells
Chen-Nen Chang2011[17]	New or Recurrent III/IV	I/II	17(8); 63(UK); historical	44.7; UK	Median; 90	CT/RT	DCs loaded with AIT (s.c)	1.0–6.1×10^7^/course	GM-CSF, IL-4
Ryuya Yamanaka 2005[18]	RecurrentIII/IV	I/II	24(16); 27(UK); historical	48.9; 55.9	Median; 62.5	SR/RT, CT	DCs loaded with ATL (i.d or i.t)	3.9–240.9×10^6^/course	GM-CSF, IL-4, KLH
Christopher J Wheeler 2004[19]	De novo IV	I/II	25(11); 25(13); randomized	55; 50	>60	SR/CT, RT	DCs loaded with ATL or HLP	10–40 ×10^6^/course	UK
Linda M.Liau 2005[20]	New or Recurrent IV	I	12(5); 99(UK); historical	40.4; <50	≥60	CT/ICH	DCs loaded with ATP	1–10×10^6^/course	GM-CSF, IL-4
Tetsuro Kikuchi 2001[21]	Recurrent UK	I	8(7)	38	Median 70	SR/CT, RT	DCs fused with AIT (i.d)	2.4–8.7×10^6^/course	GM-CSF, IL-4,TNF-α
R Yamanaka 2003[22]	Recurrent UK	I/II	10(4)	46	Median 54	SR/RT	DCs loaded with ATL (i.d)	10–137.2×10^6^/course	GM-CSF, IL-4, KLH
John S. Yu 2004[23]	New or Recurrent UK	I	14(10); 26(UK); historical	45; 53	≥60	SR/CT	DCs loaded with ATL (i.d)	10^7^–10^8^/course	GM-CSF, IL-4
X.Jie 2012[24]	Recurrent IV	I/II	13(10); 12(9); randomized	40.2; 43.1	≥60	SR/RT, CT	DCs loaded with AHT and GM-CSF(s.c)	6×10^6^/course	GM-CSF, IL-4, IL-1β, PGE2, TNF-α
Der-Yang Cho 2011[25]	New IV	II	18(8); 16(8); randomized	58.6; 55.8	>70	SR/CT, RT	DCs loaded with ATL(s.c)	2–5×10^7^/course	GM-CSF, IL-4

The table summarizes the patients' basic information about the tumor stage, newly or recurrent, cases, age, KPS, operative method before the immunotherapy and details of the immunotherapy including the DC, tumor antigen, and loading route. The last row is the culture conditions used for the cells. KPS: Karnofsky performance status; AIT: Autologous irradiated tumor cells; ATL: Autologous tumor lysate; HLP: HLA-1-eluted peptides; ATP: Autologous acid-eluted tumor peptides; AHT: Autologous heat-shock tumor cells; CT: Chemical therapy; RT: Radiation therapy; SR: Surgical resection; KLH: Keyhole limpet hemocyanin; PGE2: Prostaglandin E2; TNF-α: Tumor necrosis factor-α; IL-4: Interleukin-4; i.d.: intradermalvaccination; i.t.: intratumoral vaccination; s.c.:subcutaneous injection; ICH: intra-cellular hyperthermia; UK: Unknown.

The method for the preparation of DCs is now well established, and a sufficient number of DCs can be generated for injection into patients. In Table 1, we summarized the patient information about the DC treatment. DCs were matured using cocktails containing GM-CSF, IL-4, TNF-α, IL-1β, or PGE2. The number of DCs injected ranged from 1×10^6^ to 5×10^8^. The frequency of the injections was highly variable in different trials. The sources of antigen were also different, but most that were included in our meta-analysis were derived from tumor cells: autologous irradiated tumor cells (AIT), autologous tumor lysate (ATL), HLA-1-eluted peptides (HLP), autologous acid-eluted tumor peptides (ATP), and autologous heat-shock tumor cells (AHT). One trial reported DC treatment with fusions of glioma cells [21]. The routes of DC injection used were mainly intradermal (i.d.), intratumoral (i.t.), and subcutaneous (s.c.).

### 3.3 Survival

#### 0.5-year overall survival

**Figure 2:**
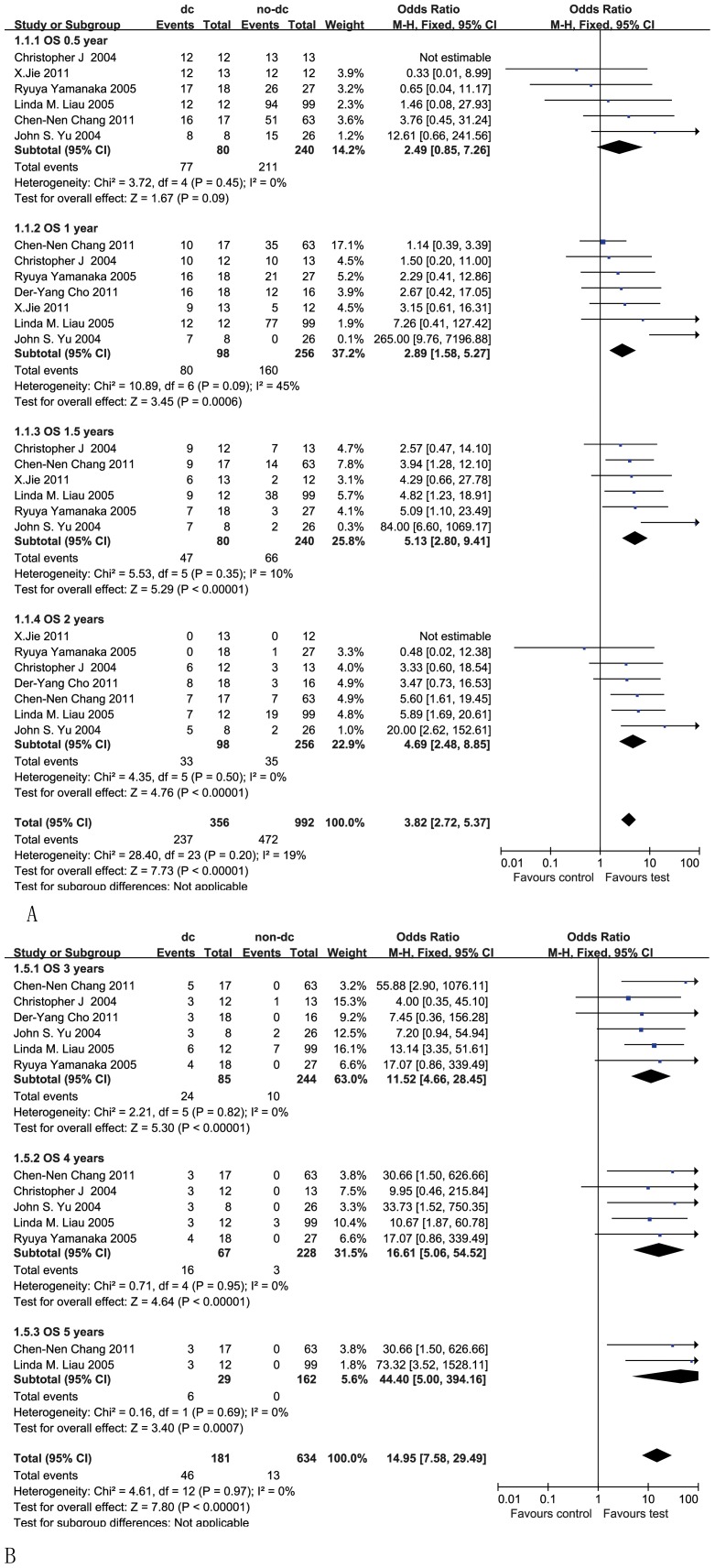
Comparison of 0.5-, 1-, 1.5- and 2-year overall survival (OS) between the non-DC and DC groups (A); Forest plot for 3-, 4-, and 5-year OS between the non-DC and DC groups in HGG patients (B). The fixed-effects meta-analysis model (Mantel-Haenszel method) was used. OR, odds ratio. DC, DC-containing therapy; non-DC, non-DC-containing therapy. Each trial is represented by a square, the center of which gives the odds ratio for that trial. The size of the square is proportional to the information in that trial. The ends of the horizontal bars denote a 95% CI. The black diamond gives the overall odds ratio for the combined results of all trials.

Information on the 0.5-year survival was available for six trials [17]–[19], [20], [23], [24]. These six trials contained 320 patients in total (80 patients received DC therapy, and 240 patients not receiving DC therapy were used as a control). Although the 0.5-year OS rates were 96% (77/80) for glioma patients receiving the DC treatment and 88% (211/240) for the historical or nonrandomized and randomized control cohorts, the estimated pooled OR for these six trials did not show a significantly improved 0.5-year OS for patients who received DC therapy compared with the non-DC therapy group (OR 2.49, 95% CI 0.85 – 7.26, *P* = 0.09). Cochran's Q test yielded a P value of 0.45, and the corresponding I^2^ quantity was 0%, indicating that the degree of variability between the trials was consistent with what would be expected to occur by chance alone (Figure 2A).

#### 1-year overall survival

Information on the 1-year survival was available for seven trials [17]–[20], [23]–[25]. These seven trials contained 354 patients in total (98 patients received DC therapy, and 256 control patients did not receive DC therapy). The 1-year overall survival rate was 82% (80/98) for glioma patients receiving the DC treatment, whereas it was 63% (160/256) for the controls. The meta-analysis showed a significantly improved 1-year OS for the patients who received DC therapy compared with those who did not (OR 2.89, 95% CI 1.58–5.27, *P* = 0.0006). Cochran's Q test yielded a P value of 0.09, and the corresponding I^2^ quantity was 45% (Figure 2A).

#### 1.5-year overall survival

Information on the 1.5-year survival was available for six trials [17]–[20], [23], [24]. These six trials contained 320 patients in total (80 patients received DC therapy, and 240 patients who did not receive DC therapy served as a control). The 1.5-year overall survival rates were 59% (47/80) for glioma patients receiving DC treatment and 28% (66/240) for controls. The meta-analysis showed a significant benefit for the 1.5-year OS in the HGG patients who received DC therapy compared with non-DC therapy (OR 5.13, 95% CI 2.80–9.41, P<0.00001). Cochran's Q test yielded a P value of 0.35, and the corresponding I2 quantity was 10% (<50%), indicating that the degree of variability between the trials was consistent with what would be expected to occur by chance alone (Figure 2A).

#### 2-year overall survival

Information on the 2-year survival was available for seven trials [17]–[20], [23]–[25]. These seven trials contained 354 patients in total (98 patients received DC therapy, and 256 patients who did not receive DC therapy served as a control). The 2-year OS rates were 34% (33/98) for glioma patients receiving DC treatment and 14% (35/256) for the controls. The estimated pooled OR for these seven trials showed a significantly increased 2-year OS for the patients who received DC therapy compared with those who did not (OR 4.69, 95% CI 2.48–8.85, *P*<0.00001). Cochran's Q test had a P value of 0.50, and the corresponding I^2^ quantity was 0% (Figure 2A).

#### 3-year overall survival

Information on the 3-year survival was available for six trials [17]–[20], [23], [25]. These six trials included 354 patients in total (98 patients received DC therapy, and 256 patients who did not receive DC therapy were used as controls). The 3-year OS rate was 24% (24/98) for glioma patients receiving DC treatment, whereas it was 4% (10/256) for the controls. The meta-analysis showed a significantly longer 3-year OS for the patients who received DC therapy compared with those who did not (OR 11.52, 95% CI 4.66–28.45, *P*<0.00001). Cochran's Q test had a P value of 0.82, and the corresponding I^2^ quantity was 0% (Figure 2B).

#### 4-year overall survival

Information on the 4-year survival was available for five trials [17]–[20], [23]. These five trials contained 320 patients in total (80 patients received DC therapy, and 240 patients who did not receive DC therapy were used as a control). The 4-year OS rates were 20% (16/80) for glioma patients receiving DC treatment and 1% (3/240) for the controls. The meta-analysis showed a significant improvement of the 4-year OS in the HGG patients who received DC therapy compared with those who did not (OR 16.61, 95% CI 5.06–54.52, *P*<0.00001). Cochran's Q test had a P value of 0.97, and the corresponding I^2^ quantity was 0% (Figure 2B).

#### 5-year overall survival

Information on the 5-year survival was available for two trials [17], [20]. These two trials contained 216 patients in total (42 patients received DC therapy, and 174 control patients did not). The 5-year OS rate was 14% (6/42) for glioma patients receiving the DC treatment, whereas it was ultimately 0% (0/174) for the controls. The meta-analysis showed a significantly greater 5-year OS for the patients who received DC therapy compared with those who did not (OR 44.40, 95% CI 5.00–394.16, *P* = 0.0007). Cochran's Q test had a P value of 0.69, and the corresponding I^2^ quantity was 0% (Figure 2B), indicating that the degree of variability between the trials was consistent with what would be expected to occur by chance alone.

#### 0.5-year progression-free survival

**Figure 3:**
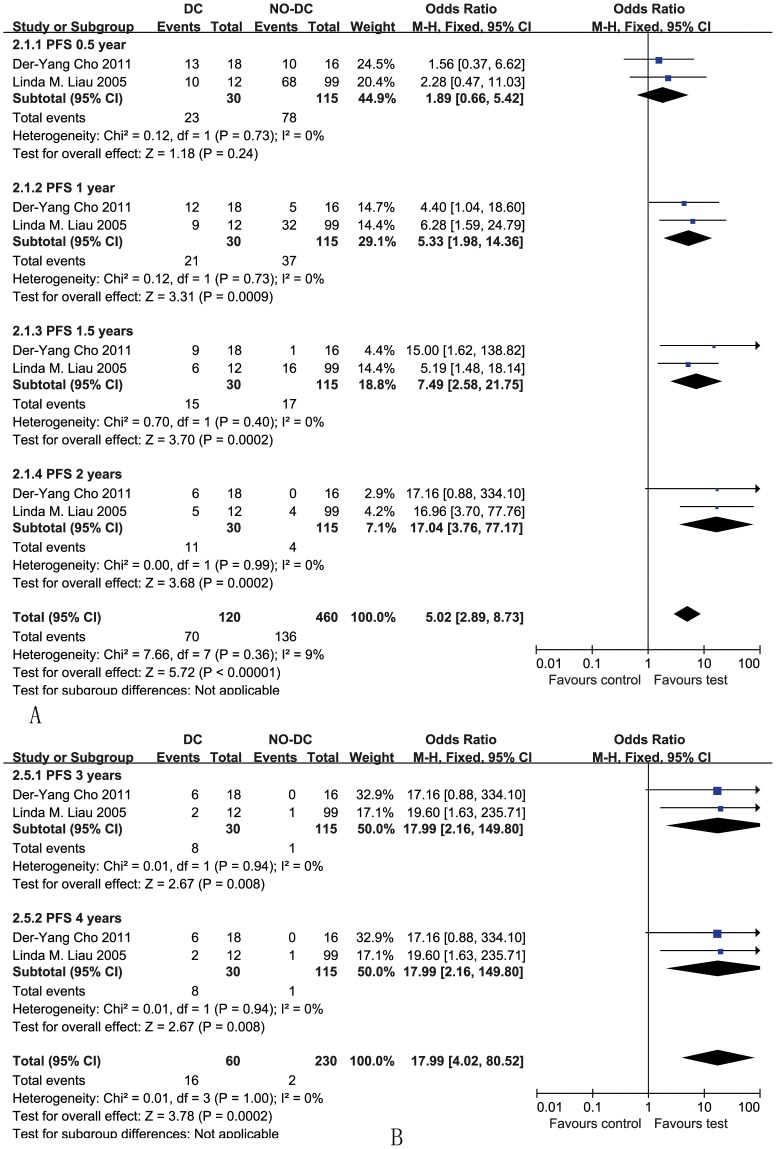
Comparison of 0.5-, 1-, 1.5-, and 2-year progression-free survival (PFS) between the non-DC and DC groups (A); Forest plot for 3-, 4-, and 5-year PFS between the non-DC and DC groups in HGG patients (B). The fixed effects meta-analysis model (Mantel-Haenszel method) was used in this analysis.

Information on the 0.5-year PFS was available for two trials [20], [25] and contained 145 patients (30 patients received DC immunotherapy) (Figure 3A). DC immunotherapy led to a 0.5-year PFS in 77% (23/30) of glioma patients. In contrast, the 0.5-year PFS was only 68% (78/115) in patients without DC immunotherapy. However, the results showed that there was no significant improvement of the 0.5-year PFS for the patients who received DC therapy compared with the non-DC therapy group (OR 1.89, 95% CI 0.66–5.42, *P* = 0.24). Cochran's Q test had a P value of 0.73, and the corresponding I^2^quantity was 0%.

#### 1-, 1.5-, and 2-year progression-free survival

Information on the 1-, 1.5-, and 2-year PFS was available for two trials [20], [25], which contained 145 patients (30 patients received DC immunotherapy) (Figure 3A). DC immunotherapy led to a 1-, 1.5-, and 2-year PFS of 70%, 50%, and 37% (21/30, 15/30 and 11/30), respectively, in HGG patients who received DC treatment, whereas the 1-, 1.5-, and 2-year PFS in the controls was only 32%, 15%, and 3% (37/115, 17/115, and 4/115), respectively. Both of the trials showed a longer disease-free survival for patients who received DC immunotherapy in comparison to the historical or randomized cohorts at one, one and a half and two years. The estimated pooled OR for the two trials showed a highly significantly improved one, one and a half, and two-year PFS for patients receiving DC immunotherapy (OR 5.33, 95% CI 1.98–14.36, *P* = 0.0009; OR 7.49, 95% CI 2.58–21.75, *P* = 0.0002; OR 17.04, 95% CI 3.76–77.17, *P* = 0.0002) (Figure 3A). The overall Cochran's Q test yielded a P value of 0.36, and the corresponding I^2^ quantity was 9% (<50%).

#### 3- and 4-year progression-free survival

Information on the 3- and 4-year PFS was available for two trials [20], [25] and contained 145 patients (30 patients received DC immunotherapy) (Figure 3B). DC immunotherapy led to a 3- and 4-year PFS of 27% (8/30) in glioma patients. In contrast, the 3- and 4-year PFS was only 1% (1/115) in patients who did not receive DC immunotherapy. Both trials showed a longer PFS for DC immunotherapy in comparison to the controls at three and four years. The estimated pooled OR for the two trials showed a highly significantly improved three- and four-year PFS for patients receiving DC immunotherapy (OR 17.99, 95% CI 2.16–149.80, *P* = 0.008). The overall Cochran's Q test had a P value of 1.00, and the corresponding I^2^ quantity was 0%.

### 3.4 Function response rate

**Figure 4:**
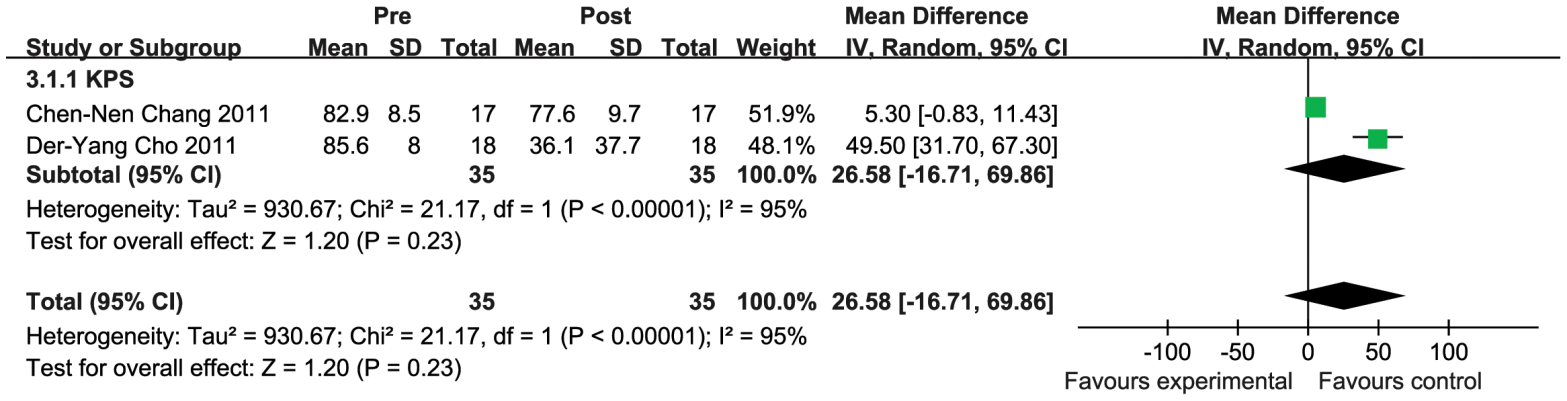
Forest plot for KPS before and after DC treatment. The random effects model (Mantel-Haenszel method) was used in this analysis.

The analysis of KPS demonstrated no favorable results for the DC cell therapy arm, with the OR being 26.58 (95% CI −16.71–69.86, *P = *0.23). The overall Cochran's Q test yielded *P*<0.00001, and the corresponding I^2^ quantity was 95% (>50%) (Figure 4).Thus the random effects model was used in this analysis and it showed that the significant heterogeneity exist between the extracted data of KPS.

### 3.5 Immune response

#### Lymphocyte/monocyte subsets in patients

**Figure 5:**
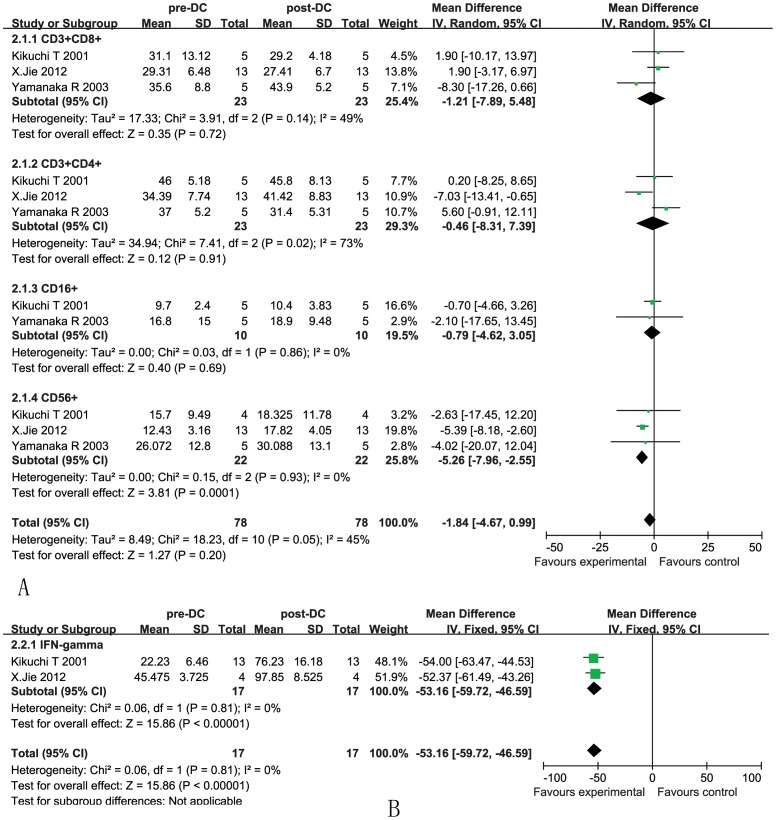
Forest plot for the immunophenotype (A) and immune cytokine (B) assessment. The data were collected from the patients before and after DC treatment. The random and fixed effects meta-analysis model (Mantel-Haenszel method) was used in this analysis.

The analysis showed that the proportions of CD3^+^CD8^+^ and CD3^+^CD4^+^ cells were not significantly increased in the DC group compared with the baseline levels observed before treatment, as reflected by pooled MD values of −1.21 (95% CI = −7.89–5.48, *p* = 0.72) and −0.46 (95% CI = −8.31–7.39, *p* = 0.91) [21], [22], [24]. Cochran's Q test had P values of 0.14 and 0.02, while the corresponding I^2^ quantities were 49% and 73%. CD16^+^ cells were also not significantly increased in the DC group compared with the baseline levels observed before treatment, as reflected by pooled MD values of −0.79 (95% CI = −4.62–3.05, *p* = 0.69), cochran's Q test had a P value of 0.86, while the corresponding I^2^ quantity was 0%. Whereas CD56^+^ lymphocyte subset was significantly increased after DC treatment with pooled MD values of −5.26 (95% CI = −7.96–−2.55, *p* = 0.0001) (Figure 5A). Cochran's Q test had a P value of 0.93, while the corresponding I^2^ quantity was 0%.

#### Immune cytokine levels of patients

As a consequence of stimulation by DCs, the CD4^+^ cells release cytokines, such as IL-2, IL-6, IFN-γ, TNF, and lymphotoxin (LT), which assist in the expansion of the CD8^+^ cytotoxic T lymphocytes (CTLs). In our analysis, the levels of IFN-γ (OR = -53.16, 95% CI = −59.72–−46.59, *p*<0.00001) were significantly increased after DC treatment (Figure 5B) [21], [24]. Cochran's Q test had a P value of 0.41, while the corresponding I^2^ quantity was 0%, indicating that there was no evidence of heterogeneity among the individual studies. This finding indicated that the degree of variability among the trials was consistent with what would occur by chance.

## Discussion

DC therapy is based on the concept that GBM cells are poor APCs because of the down regulation of costimulatory molecules and the release of immunoinhibitory cytokines [6]. DCs are professional APCs that phagocytose foreign antigens and present them in the context of MHC to activate innate and adaptive immune cells [8]. Thus, DC immunotherapy is widely considered as the fourth treatment modality for patients with cancer [26].

Our systematic meta-analysis yielded several major findings. First, we demonstrated that DC immunotherapy can significantly improve the 1-, 1.5-, 2-, 3-, 4-, and 5-year OS (*p*<0.001) of HGG patients compared with the non-treatment group. Then, a meta-analysis of the outcome of the patient data revealed that DC immunotherapy has a significant influence on the 1-, 1.5-, 2-, 3-, and 4-year PFS (*p*<0.001). But, the results of the analysis of KPS demonstrated no favorable outcome for DC cell therapy arm (*p = *0.23). Furthermore, the percentages of CD3^+^CD8^+^ and CD3^+^CD4^+^ T cells and CD16^+^ lymphocyte cells were not significantly increased in the DC group compared with the baseline levels observed before treatment (*p*>0.05), but CD56^+^ lymphocyte cells were significantly increased after DC treatment (*p*<0.001). In addition, after DC immunotherapy, the levels of IFN-γ in the peripheral blood of HGG patients, which reflect the immune function of the patients, were significantly increased (*p*<0.001). Overall, according to our analysis, DC therapy can prolong OS, improve the disease recurrence time, and would involve in the immunity function. Hence, our meta-analysis demonstrated that DC immunotherapy is a promising therapy method for HGG patients.

To date, most recent phase I and II trials have exhibited the median overall survival is increased by 20% after administration of an autologous DC vaccine for patients with GBM [17], [27]. Thus, in a meta-analysis of the collected data, our comprehensive results showed that the 1-, 1.5-, 2-, 3-, 4-, and 5-year OS rates were 82%, 59%, 34%, 24%, 20%, and 14%, respectively, which is slightly different from the results observed with the independent trials and the median overall survival was about 29%. Yet, the positive trend held consistent compared with the historical or the randomized and nonrandomized controls, which maintained 63%, 28%, 14%, 4%, 1%, and 0 OS rates, respectively and the median overall survival was about 9%. So through logistic regression, our data analysis also showed that DC immunotherapy can significantly prolong the OS in HGG patients (*p*<0.001) by increasing median OS 20%.

Regarding PFS, the data were described in only two studies of 145 patients with historical cohorts or randomized controls. The summarized results showed that the 1-, 1.5-, 2-, 3-, and 4-year PFS rates were 70%, 50%, 37%, and 27%, respectively, compared with the controls, which maintained 32%, 15%, 3%, and 1% PFS rates. Our meta-analysis showed that DC immunotherapy benefits the PFS, which could be up to 50% at the 1.5-year mark. It was previously reported that independent clinical trials of Phase II studies with other immunotherapy methods in recurrent GBM showed that the median PFS is 20 weeks [28], and in another Phase II trial with newly diagnosed GBM, the time for the median PFS is 14.2 months [29]. Although there are quite a few differences among the trials, the positive trend of the meta-analysis was fully confirmed, and the advantage of logistic regression for the data analysis was obvious, revealing that DC immunotherapy has a significant influence on the 1-, 1.5-, 2-, 3-, and 4-year PFS (*p*<0.001). As a matter of fact, immunotherapy would ameliorate some of the symptoms: patients had increased appetite, improved sleep, gained body weight, and pain relief. But in our meta-analysis, DC immunotherapy may not improve the life quality of postoperative patients (*p* = 0.23) by comparing KPS before and after treatment on the Figure 4.

Immunologic evidence of the response to DC therapy was assessed by comparing the levels of immunologic cell types (CD3^+^CD8^+^, CD4^+^CD8^+^, CD16^+^ and CD56^+^ cells) and certain cytokines (IFN-γ) before and after DC treatment. Our meta-analysis showed that following DC therapy, there was a significant increase in the IFN-γ (*p*<0.001) levels compared with those in non-DC patients, suggesting the induction of an immune response in these patients with DC treatment. The concept of immune-editing is to use immunotherapy as a treatment strategy in response to a major challenge presented to the immune system [30]. Immune-editing consists of 3 phases: elimination, equilibrium, and escape. Elimination refers to the antitumor function of both the adaptive and innate immune system and is driven by the production of IFN-γ. It was demonstrated that IFN-γ production levels from post-vaccine peripheral blood mononucleated cell (PBMC) correlated significantly with patient survival and time to progression [31]. Here, our meta-analysis demonstrated that IFN-γ was significantly increased after DC treatment and thus would be helpful in immune-editing to compensate for the elimination. But it should be noted that only two trials were included, and the method for measure IFN-γ was not used ELISPOT and/or ICS. Furthermore, in the included studies, there were some trials in which they detected the IFN-γ with ELISPOT only reported the individual response of the patients [18], [22] or qPCR to calculate the multiples or the percentage of the response after the immunotherapy [19], [23], but could not be analyzed in our meta-analysis. Thus the quantification of IFN-γ in sera was not consistent in all the studies, that could induce the big heterogeneity among these studies.

Another notable challenge is the presence of an immunosuppressive tumor microenvironment, which causes decreased antigen recognition and depressed immune cell activation. Although increased CD8^+^ infiltrating lymphocytes have been shown in some studies to be associated with increased patient survival, Hussain et al. reported that most tumor-infiltrating CD8^+^ cells are not activated [2], [32]. In our meta-analysis, the results showed that the CD3^+^CD8^+^ and CD4^+^CD8^+^ levels were not significantly changed after DC treatment. In addition, our meta-analysis that included only three trials showed CD16^+^ and CD56^+^ lymphocyte cells which would denote some of the NK cells, could combat the tumor cells and play an immunomodulatory function, inducing a Th1 immune response and improving antitumor immunoreactivity in the body [33]. Among them CD16^+^ was not significantly increased after DC treatment, but CD56^+^ was significantly increased after DC immunotherapy according to our meta-analysis with the included papers.

Equilibrium is the period in which immune cells become latent to a partially eradicated tumor. Escape occurs when the tumor escapes from immunosurveillance and becomes resistant to antitumor immune function, usually via genomic instability or downregulation of key antigens [34]. Thus, to induce a tumor-specific immune reaction via DCs, which is the basis of DC immunotherapy, DCs are loaded with various antigens and then activate both helper and killer T cells and B cells. So far, the different tumor-associated antigens (TAAs) used include specific tumor-associated peptides, tumor-derived RNA and cDNA, tumor cell lysate, apoptotic tumor cells, and gene transfer methods using retroviral vectors, recombinant adenoviruses or lentiviruses encoding tumor antigens, and electroporation of tumor RNA into DCs to increase the target accuracy and overcome the tumor immunosuppression [35]. In our meta-analysis, the selected clinical trials with DC only used the following tumor antigens: autologous irradiated tumor cells, tumor lysate and acid-eluted tumor peptides or heat-shock tumor cells that compensate for tumor antigen exposure. Other antigens were not included in our meta-analysis, especially EGFRvIII, IL-13Rα, EphA2, survivin, Wilms' tumor 1 (WT1), Sry-related high mobility group box (SOX), and cytomegalovirus (CMV), but have been tested in some clinical trials [36]. Furthermore, cancer stem cells (CSCs) or cancer-initiating cells can also be a potentially useful source of tumor antigens in DC-based immunotherapy, and some of the preclinical data are potentially encouraging [37]. Thus, multiple questions need to be clarified regarding the identification of suitable antigens and improvement of the tumor cell targeting accuracy precluding the eventually successful translation of DC immunotherapy into clinical applications.

More recent transcriptional profiling has classified GBM molecularly into four subtypes with distinct clinical features. Prins et al. showed the difference of sensitivity to a whole-cell lysate DC vaccination between two of the GBM subtypes with MGMT promoter methylation and IDH1 mutation [38]. Interestingly, it was also demonstrated that the mesenchymal gene expression profile may represent a population of patients with favorable responses to their DC vaccine, so the GBM microenvironment is also a double-edged sword concerning DC immunotherapy [39]. Despite these challenges, DC immunotherapy still showed an appealing benefit in terms of extending the survival of patients with HGG. The heterogeneous methods use and the complexity of designing and reporting on the immunotherapy trials will be overcome in the near future, providing stronger evidence to establish DC treatment as the standard for HGG patients.

In brief, DC immunotherapy has yielded encouraging results with immunological and clinical benefits for HGG patients and needs to be further tested to demonstrate significant therapeutic efficacy in phase III clinical trials.

### Limitations of the study

Although several early-phase clinical trials have demonstrated promising therapeutic outcomes to date, clinical immunotherapy trials for gliomas have not yet demonstrated objective proof of clinical trials for lacking the randomized studies, so limitations in our analyses should be considered in interpreting the results. Many intrinsic and extrinsic factors might influence the systemic review's reliability. First, no one trial has more than 100 patients per arm. Therefore, there is a lack of multinational, large-samples, multicenter clinical trials regarding DC cell therapy for HGG. Second, not all of the included studies reported clinical random allocation concealment, and in most cases, we collected data from the nonrandomized and randomized control or historical cohorts; thus, distribution and implementation biases may exist in our meta-analysis. Moreover, the analysis performed in this study was not based on individual patient data and was not subjected to an open external evaluation procedure. Maybe here one should refer to the proposed HGG-IMMUNO RPA model for use in future reports for relapsed patients treated with immunotherapy [11]. In addition, to maintain consistency during our systematic review, we selected only assessable patients for our analysis. These sampling factors may also introduce bias into our conclusions, for example considering the age-, degree of resection and therapy, gender- and disease-matched controls, and thus our analysis may have led to an overestimation of the treatment effects. However, we expect that our study will be valuable for the design of more comprehensive, larger, controlled clinical trials.

Furthermore, the blood sample sources, injection modes, cell numbers, cell purity, tumor antigen, and cell phenotype may also affect the outcome of individual trials. All of these variables may introduce some level of bias; for instance, there is significant heterogeneity in the extracted data shown in Figure 4 and 5. It is important to standardize not only the DC cell preparation but also the criteria of the immune phenotyping system and the clinical response assessment. Our analysis may be valuable for the standardization of DC immune therapy as an adjuvant treatment for patients with HGG.

## Conclusions

Taken together, our data suggest that DC cells have great potential to be a clinically efficacious therapy in the treatment of patients suffering from advanced-stage HGG malignancies who exhibit poor tolerance of chemotherapy or radiotherapy. These early results from clinical trials are very promising and must be verified more stringently before DC immunotherapy can be applied at the bedside.

## Supporting Information

Checklist S1 PRISMA Checklist.
(DOC)

File S1 Contains Tables S1–S7. **Table S1**. List 9 excluding papers for reviews. **Table S2**. List 11 excluding papers for being in vitro experiments. **Table S3**. List 26 excluding papers for being animal models experiments. **Table S4**. List 91 case reports for being no enough data. **Table S5**. List 21 DC protocol studies and comments for being no clinical data. **Table S6**. List 22 clinical trials for no appropriate control arm. Specify the reasons of excluded clinical trial study's PICOS characteristics and report characteristics. P: participants; I: interventions; C: comparison; O: outcomes; S: setting (study design). **Table S7**. List 9 including clinical trials. Separately specify all the selected information sources in the included 9 clinical trials for our meta-analysis with PICOS, report characteristics, and clinical data source which denoted every data extracted from the paper's Title, Abstract, Introduction, Material and methods, Results, Figure, Table, Discussion, or Supplementary material.
(DOCX)
