# Supplementary material for: Clinical Efficacy of Tumor Antigen-Pulsed DC Treatment for High-Grade Glioma Patients: Evidence from a Meta-Analysis
Source: PLoS One. 2014 Sep 12;9(9):e107173. doi: 10.1371/journal.pone.0107173 (PMC4162602; doi:10.1371/journal.pone.0107173)
Supplement: File S1 — Contains Tables S1–S7. Table S1. List 9 excluding papers for reviews. Table S2. List 11 excluding papers for being in vitro experiments. Table S3. List 26 excluding papers for being animal models experiments. Table S4. List 91 case reports for being no enough data. Table S5. List 21 DC protocol studies and comments for being no clinical data. Table S6. List 22 clinical trials for no appropriate control arm. Specify the reasons of excluded clinical trial study's PICOS characteristics and report characteristics. P: participants; I: interventions; C: comparison; O: outcomes; S: setting (study design). Table S7. List 9 including clinical trials. Separately specify all the selected information sources in the included 9 clinical trials for our meta-analysis with PICOS, report characteristics, and clinical data source which denoted every data extracted from the paper's Title, Abstract, Introduction, Material and methods, Results, Figure, Table, Discussion, or Supplementary material. (DOCX) [file pone.0107173.s002.docx]

**Table 1 List 9 excluding papers for reviews**

| **Excluding 9 reviews for no clinical data** |
| --- |
| 1. Marsh JC, Goldfarb J, Shafman TD, Diaz AZ. Current status of immunotherapy and gene therapy for high-grade gliomas. Cancer Control. 2013 Jan;20(1):43-8. Review. PMID: 23302906. |
| 2. Mineharu Y, Castro MG, Lowenstein PR, Sakai N, Miyamoto S. Dendritic cell-based immunotherapy for glioma: multiple regimens and implications in clinical trials. Neurol Med Chir (Tokyo). 2013;53(11):741-54. PMID: 24140772. |
| 3. Kuijlen JM, Bremer E, Mooij JJ, den Dunnen WF, Helfrich W. Review: on TRAIL for malignant glioma therapy? Neuropathol Appl Neurobiol. 2010 Apr;36(3):168-82. doi: 10.1111/j.1365-2990.2010.01069.x. PMID: 20102513. |
| 4. Mehrpour M, Codogno P. Prion protein: From physiology to cancer biology. Cancer Lett. 2010 Apr 1; 290(1):1-23. doi: 10.1016/j.canlet.2009.07.009. PMID: 19674833. |
| 5. Xu X, Stockhammer F, Schmitt M. Cellular-based immunotherapies for patients with glioblastoma multiforme. Clin Dev Immunol. 2012;2012:764213. doi: 10.1155/2012/764213. Review. PMID: 22474481; |
| 6. Panner A, Parsa AT, Pieper RO. Use of APO2L/TRAIL with mTOR inhibitors in the treatment of glioblastoma multiforme. Expert Rev Anticancer Ther. 2006 Sep;6(9):1313-22. PMID: 17020463. |
| 7. Shah K, Hsich G, Breakefield XO. Neural precursor cells and their role in neuro-oncology. Dev Neurosci. 2004 Mar-Aug; 26(2-4):118-30. PMID: 15711055. |
| 8. Roth W, Weller M. Chemotherapy and immunotherapy of malignant glioma: molecular mechanisms and clinical perspectives. Cell Mol Life Sci. 1999 Oct 30;56(5-6):481-506. PMID: 11212300. |
| 9. Weller M, Kleihues P, Dichgans J, Ohgaki H. CD95 ligand: lethal weapon against malignant glioma? Brain Pathol. 1998 Apr; 8(2):285-93. PMID: 9546287. |

**Table 2 List 11 excluding papers for being in vitro experiments**

| **Excluding 11 published in vitro experiments papers for no clinical data** |
| --- |
| 1. Ji B, Chen Q, Liu B, Wu L, Tian D, Guo Z, Yi W. Glioma stem cell-targeted dendritic cells as a tumor vaccine against malignant glioma. Yonsei Med J. 2013 Jan 1;54(1):92-100. doi: 10.3349/ymj.2013.54.1.92. PMID: 23225804. |
| 2. Kalthur G, Kumar P, Devi U, Ali S, Upadhya R, Pillai S, Rao A. Susceptibility of peripheral lymphocytes of brain tumour patients to in vitro radiation-induced DNA damage, a preliminary study. Clin Exp Med. 2008 Sep; 8(3):147-50. doi: 10.1007/s10238-008-0171-1. PMID: 18791687. |
| 3. Candolfi M, Pluhar GE, Kroeger K, Puntel M, Curtin J, Barcia C, Muhammad AK, Xiong W, Liu C, Mondkar S, Kuoy W, Kang T, McNeil EA, Freese AB, Ohlfest JR, Moore P, Palmer D, Ng P, Young JD, Lowenstein PR, Castro MG. Optimization of adenoviral vector-mediated transgene expression in the canine brain in vivo, and in canine glioma cells in vitro. Neuro Oncol. 2007 Jul; 9(3):245-58. PMID: 17522335. |
| 4. Rooprai HK, Kyriazis I, Nuttall RK, Edwards DR, Zicha D, Aubyn D, Davies D, Gullan R, Pilkington GJ. Inhibition of invasion and induction of apoptosis by selenium in human malignant brain tumour cells in vitro. Int J Oncol. 2007 May;30(5):1263-71. PMID: 17390030. |
| 5. Yamashita J, Handa H, Tokuriki Y, Ha YS, Otsuka SI, Suda K, Taki W. Intra-arterial ACNU therapy for malignant brain tumors. Experimental studies and preliminary clinical results. J Neurosurg. 1983 Sep; 59(3):424-30. PMID:6577143. |
| 6. Brooks WH, Roszman TL, Rogers AS. Impairment of rosette-forming T lymphocytes in patients with primary intracranial tumors. Cancer. 1976 Apr; 37(4):1869-73. PMID: 769940. |
| 7. Mungyerová G, Jacz K, Kuzma I, Babusiková O, Kalafut F. Experiences with malignant brain tumours in clinic and in tissue culture. Acta Neurochir (Wien). 1965;13(3):393-406. PMID: 4286798. |
| 8. Tang XJ, Lu JT, Tu HJ, Huang KM, Fu R, Cao G, Huang M, Cheng LH, Dai LJ, Zhang L. TRAIL-engineered bone marrow-derived mesenchymal stem cells: TRAIL expression and cytotoxic effects on C6 glioma cells. Anticancer Res. 2014 Feb;34(2):729-34. PMID: 24511006. |
| 9. Yulyana Y, Endaya BB, Ng WH, Guo CM, Hui KM, Lam PY, Ho IA. Carbenoxolone enhances TRAIL-induced apoptosis through the upregulation of death receptor 5 and inhibition of gap junction intercellular communication in human glioma. Stem Cells Dev. 2013 Jul 1; 22(13):1870-82. doi: 10.1089/scd.2012.0529. PMID: 23428290. |
| 10. Morgan RA, Johnson LA, Davis JL, Zheng Z, Woolard KD, Reap EA, Feldman SA, Chinnasamy N, Kuan CT, Song H, Zhang W, Fine HA, Rosenberg SA. Recognition of glioma stem cells by genetically modified T cells targeting EGFRvIII and development of adoptive cell therapy for glioma. Hum Gene Ther. 2012 Oct;23(10):1043-53. doi: 10.1089/hum.2012.041. PMID: 22780919. |
| 11. Tamura K, Wakimoto H, Agarwal AS, Rabkin SD, Bhere D, Martuza RL, Kuroda T, Kasmieh R, Shah K. Multimechanistic tumor targeted oncolytic virus overcomes resistance in brain tumors. Mol Ther. 2013 Jan;21(1):68-77. doi: 10.1038/mt.2012.175.PMID: 22929661. |

**Table 3 List 26 excluding papers for being animal models experiments**

| **Excluding 26 published animal model experiments papers for no clinical data** |
| --- |
| 1. Kim SM, Woo JS, Jeong CH, Ryu CH, Jang JD, Jeun SS. Potential application of temozolomide in mesenchymal stem cell-based TRAIL gene therapy against malignant glioma. Stem Cells Transl Med. 2014 Feb; 3(2):172-82. doi: 10.5966/sctm.2013-0132.PMID: 24436439. |
| 2. Weber TG, Pöschinger T, Galbán S, Rehemtulla A, Scheuer W. Noninvasive monitoring of pharmacodynamics and kinetics of a death receptor 5 antibody and its enhanced apoptosis induction in sequential application with doxorubicin. Neoplasia. 2013 Aug; 15(8):863-74. PMID: 23908588. |
| 3. Burton TR, Henson ES, Azad MB, Brown M, Eisenstat DD, Gibson SB. BNIP3 acts as transcriptional repressor of death receptor-5 expression and prevents TRAIL-induced cell death in gliomas. Cell Death Dis. 2013 Apr 11; 4:e587. doi: 10.1038/cddis.2013.100. PMID: 23579274. |
| 4. Hunn MK, Farrand KJ, Broadley KW, Weinkove R, Ferguson P, Miller RJ, Field CS, Petersen T, McConnell MJ, Hermans IF. Vaccination with irradiated tumor cells pulsed with an adjuvant that stimulates NKT cells is an effective treatment for glioma. Clin Cancer Res. 2012 Dec 1;18(23):6446-59. doi: 10.1158/1078-0432.CCR-12-0704. PMID: 23147997. |
| 5. Martinez-Quintanilla J, Bhere D, Heidari P, He D, Mahmood U, Shah K. Therapeutic efficacy and fate of bimodal engineered stem cells in malignant brain tumors. Stem Cells. 2013 Aug; 31(8):1706-14. doi: 10.1002/stem.1355. PMID: 23389839. |
| 6. Bo Y, Guo G, Yao W. MiRNA-mediated tumor specific delivery of TRAIL reduced glioma growth. J Neurooncol. 2013 Mar; 112(1):27-37. doi: 10.1007/s11060-012-1033-y.PMID: 23338605. |
| 7. van de Water JA, Bagci-Onder T, Agarwal AS, Wakimoto H, Roovers RC, Zhu Y, Kasmieh R, Bhere D, Van Bergen en Henegouwen PM, Shah K. Therapeutic stem cells expressing variants of EGFR-specific nanobodies have antitumor effects. Proc Natl Acad Sci U S A. 2012 Oct 9; 109(41):16642-7.  doi: 10.1073/pnas.1202832109. PMID: 23012408. |
| 8. Kim SM, Woo JS, Jeong CH, Ryu CH, Lim JY, Jeun SS. Effective combination therapy for malignant glioma with TRAIL-secreting mesenchymal stem cells and lipoxygenase inhibitor MK886. Cancer Res. 2012 Sep 15; 72(18):4807-17. doi: 10.1158/0008-5472.CAN-12-0123.PMID: 22962275. |
| 9. Chiu TL, Wang MJ, Su CC. The treatment of glioblastoma multiforme through activation of microglia and TRAIL induced by rAAV2-mediated IL-12 in a syngeneic rat model. J Biomed Sci. 2012 Apr 22; 19:45. doi: 10.1186/1423-0127-19-45.PMID: 22520731. |
| 10. Li X, Mao Q, Wang D, Zhang W, Xia H. A fiber chimeric CRAd vector Ad5/11-D24 double-armed with TRAIL and arresten for enhanced glioblastoma therapy. Hum Gene Ther. 2012 Jun; 23(6):589-96. doi: 10.1089/hum.2011.130. PMID: 22136065. |
| 11. Li J, Gu B, Meng Q, Yan Z, Gao H, Chen X, Yang X, Lu W. The use of myristic acid as a ligand of polyethylenimine/DNA nanoparticles for targeted gene therapy of glioblastoma. Nanotechnology. 2011 Oct 28; 22(43):435101.  doi: 10.1088/0957-4484/22/43/435101.PMID: 21955528. |
| 12. Qi L, Bellail AC, Rossi MR, Zhang Z, Pang H, Hunter S, Cohen C, Moreno CS, Olson JJ, Li S, Hao C. Heterogeneity of primary glioblastoma cells in the expression of caspase-8 and the response to TRAIL-induced apoptosis. Apoptosis. 2011 Nov;16(11):1150-64. doi: 10.1007/s10495-011-0645-6. PMID: 21877214. |
| 13. Balyasnikova IV, Ferguson SD, Han Y, Liu F, Lesniak MS. Therapeutic effect of neural stem cells expressing TRAIL and bortezomib in mice with glioma xenografts. Cancer Lett. 2011 Nov 28; 310(2):148-59. doi: 10.1016/j.canlet.2011.06.029.PMID: 21802840. |
| 14. Dorsey F, Mintz A, Tian X, Dowling ML, Plastaras JP, Dicker DT, Kao GD, El-Deiry WS. Tumor necrosis factor-related apoptosis-inducing ligand (TRAIL) and paclitaxel have cooperative in vivo effects against glioblastoma multiforme cells. Mol Cancer Ther. 2009 Dec;8(12):3285-95. doi: 10.1158/1535-7163.MCT-09-0415.PMID: 19996278. |
| 15. Menon LG, Kelly K, Yang HW, Kim SK, Black PM, Carroll RS. Human bone marrow-derived mesenchymal stromal cells expressing S-TRAIL as a cellular delivery vehicle for human glioma therapy. Stem Cells. 2009 Sep;27(9):2320-30.  doi: 10.1002/stem.136.PMID: 19544410. |
| 16. Horita H, Thorburn J, Frankel AE, Thorburn A. EGFR-targeted diphtheria toxin stimulates TRAIL killing of glioblastoma cells by depleting anti-apoptotic proteins. J Neurooncol. 2009 Nov;95(2):175-84. doi: 10.1007/s11060-009-9914-4.PMID: 19449148. |
| 17. Uzzaman M, Keller G, Germano IM. In vivo gene delivery by embryonic-stem-cell-derived astrocytes for malignant gliomas. Neuro Oncol. 2009 Apr;11(2):102-8. doi: 10.1215/15228517-2008-056. PMID: 18676359 . |
| 18. Tsurushima H, Yuan X, Dillehay LE, Leong KW. Radiation-inducible caspase-8 gene therapy for malignant brain tumors. Int J Radiat Oncol Biol Phys. 2008 Jun 1;71(2):517-25. doi: 10.1016/j.ijrobp.2008.02.002.  PMID: 18407431. |
| 19. Wohlfahrt ME, Beard BC, Lieber A, Kiem HP. A capsid-modified, conditionally replicating oncolytic adenovirus vector expressing TRAIL Leads to enhanced cancer cell killing in human glioblastoma models. Cancer Res. 2007 Sep 15;67(18):8783-90. PMID: 17875719. |
| 20. Braeuninger S, Chamaon K, Kropf S, Mawrin C, Wiedemann FR, Hartig R, Schoeler S, Dietzmann K, Kirches E. Short incubation with 2-methoxyestradiol kills malignant glioma cells independent of death receptor 5 upregulation. Clin Neuropathol. 2005 Jul-Aug;24(4):175-83. PMID: 16033134. |
| 21. Brower V. Search and destroy: recent research exploits adult stem cells' attraction to cancer. J Natl Cancer Inst. 2005 Mar 16;97(6):414-6. PMID:15770001. |
| 22. Saito R, Bringas JR, Panner A, Tamas M, Pieper RO, Berger MS, Bankiewicz KS. Convection-enhanced delivery of tumor necrosis factor-related apoptosis-inducing ligand with systemic administration of temozolomide prolongs survival in an intracranial glioblastoma xenograft model. Cancer Res. 2004 Oct 1;64(19):6858-62. PMID: 15466173. |
| 23. Fulda S, Wick W, Weller M, Debatin KM. Smac agonists sensitize for Apo2L/TRAIL- or anticancer drug-induced apoptosis and induce regression of malignant glioma in vivo. Nat Med. 2002 Aug;8(8):808-15.PMID: 12118245. |
| 24. Roth W, Isenmann S, Naumann U, Kügler S, Bähr M, Dichgans J, Ashkenazi A, Weller M. Locoregional Apo2L/TRAIL eradicates intracranial human malignant glioma xenografts in athymic mice in the absence of neurotoxicity. Biochem Biophys Res Commun. 1999 Nov 19;265(2):479-83. PMID: 10558893. |
| 25. Roth W, Grimmel C, Rieger L, Strik H, Takayama S, Krajewski S, Meyermann R, Dichgans J, Reed JC, Weller M. Bag-1 and Bcl-2 gene transfer in malignant glioma: modulation of cell cycle regulation and apoptosis. Brain Pathol. 2000 Apr;10(2):223-34. PMID: 10764042. |
| 26. Nagane M, Pan G, Weddle JJ, Dixit VM, Cavenee WK, Huang HJ. Increased death receptor 5 expression by chemotherapeutic agents in human gliomas causes synergistic cytotoxicity with tumor necrosis factor-related apoptosis-inducing ligand in vitro and in vivo. Cancer Res. 2000 Feb 15;60(4):847-53. PMID: 10706092. |

**Table 4 List 91 case reports for being no enough data**

| **Excluding 91 published case reports for no clinical data** |
| --- |
| 1. Chi AS, Chamberlain MC. Is there a role for bevacizumab in the treatment of glioblastoma? Oncologist. 2013;18(10):1080-2. doi:10.1634/theoncologist.2013-0296.PMID: 24056893. |
| 2. Phillips J, East HE, French SE, Melcescu E, Hamilton RD, Nicholas WC, Fratkin JF, Parent AD, Luzardo G, Koch CA. What causes a prolactinoma to be aggressive or to become a pituitary carcinoma? Hormones (Athens). 2012 Oct-Dec;11(4):477-82. PMID: 23422771. |
| 3. Serebruany VL, Dinicolantonio JJ, Can MM, Goto S. Unclassified pleomorphic and spindle cell pulmonary neoplasm with brain metastases after prasugrel. Cardiology. 2013;124(2):85-90. doi: 10.1159/000346382. PMID: 23369842. |
| 4. Villano JL, Williams LE, Watson KS, Ignatius N, Wilson MT, Valyi-Nagy T, Michals EA, Engelhard HH. Delayed response and survival from NovoTTF-100A in recurrent GBM. Med Oncol. 2013 Mar;30(1):338. doi: 10.1007/s12032-012-0338-1. PMID: 23307238. |
| 5. McDonald CR, White NS, Farid N, Lai G, Kuperman JM, Bartsch H, Hagler DJ, Kesari S, Carter BS, Chen CC, Dale AM. Recovery of white matter tracts in regions of peritumoral FLAIR hyperintensity with use of restriction spectrum imaging. AJNR Am J Neuroradiol. 2013 Jun-Jul;34(6):1157-63. doi: 10.3174/ajnr.A3372. PMID: 23275591. |
| 6. Anderson RC, Kennedy B, Yanes CL, Garvin J, Needle M, Canoll P, Feldstein NA, Bruce JN. Convection-enhanced delivery of topotecan into diffuse intrinsic brainstem tumors in children. J Neurosurg Pediatr. 2013 Mar;11(3):289-95. doi:10.3171/2012.10.PEDS12142. PMID: 23240851. |
| 7. Felix FH, de Araujo OL, da Trindade KM, Trompieri NM, Fontenele JB. Survival of children with malignant brain tumors receiving valproate: a retrospective study. Childs Nerv Syst. 2013 Feb;29(2):195-7. doi: 10.1007/s00381-012-1997-0.PMID: 23233213. |
| 8. von Campe G, Moschopulos M, Hefti M. 5-Aminolevulinic acid-induced protoporphyrin IX fluorescence as immediate intraoperative indicator to improve the safety of malignant or high-grade brain tumor diagnosis in frameless stereotactic biopsies. Acta Neurochir (Wien). 2012 Apr; 154(4):585-8; discussion 588. doi: 10.1007/s00701-012-1290-8. PMID: 22297399. |
| 9. Müller K, Zwiener I, Welker H, Maass E, Bongartz R, Berthold F, Pietsch T, Warmuth-Metz M, von Bueren A, Rutkowski S. Curative treatment for central nervous system medulloepithelioma despite residual disease after resection. Report of two cases treated according to the GPHO Protocol HIT 2000 and review of the literature. Strahlenther Onkol. 2011 Nov;187(11):757-62. doi:10.1007/s00066-011-2256-0. PMID: 22037651. |
| 10. Simal-Julián JA, Sanchis-Martín R, Prat-Acín R, Miranda-Lloret P, Conde-Sardón R, Cárdenas-Ruiz-Valdepeñas E, Beltrán-Giner A. Spinal pleomorphic xantoastrocytoma. Case report. Neurocirugia (Astur). 2010 Oct;21(5):390-5. PMID: 21042690. |
| 11. Balossier A, Dörner L, Emery E, Heese O, Mehdorn HM, Menei P, Singh J. Incorporating BCNU wafers into malignant glioma treatment: European case studies. Clin Drug Investig. 2010;30(3):195-204. doi: 10.2165/11532900-000000000-00000.PMID: 20155992. |
| 12. Michael BD, Syndikus I, Clark A, Baborie A. Diffuse primary leptomeningeal melanocytosis in a patient receiving a novel cancer cell vaccine for prostate cancer. BMJ Case Rep. 2010 May 4; doi:10.1136/bcr.11.2009.2495.PMID: 22736604. |
| 13. Shibahara I, Kumabe T, Kanamori M, Saito R, Sonoda Y, Watanabe M, Iwata R, Higano S, Takanami K, Takai Y, Tominaga T. Imaging of hypoxic lesions in patients with gliomas by using positron emission tomography with 1-(2-[18F] fluoro-1-[hydroxymethyl]ethoxy)methyl-2-nitroimidazole, a new 18F-labeled 2-nitroimidazole analog. J Neurosurg. 2010 Aug;113(2):358-68. doi:10.3171/2009.10.JNS09510.PMID: 19895196. |
| 14. Neuloh G, Schramm J. Are there false-negative results of motor evoked potential monitoring in brain surgery? Cent Eur Neurosurg. 2009 Nov;70(4):171-5. PMID: 19851956. |
| 15. Bien E, Stachowicz-Stencel T, Szalewska M, Krawczyk M, Synakiewicz A, Dubaniewicz-Wybieralska M, Zielinski P, Adamkiewicz-Drozynska E, Balcerska A. Poor-risk high-grade gliomas in three survivors of childhood acute lymphoblastic leukaemia--an overview of causative factors and possible therapeutic options. Childs Nerv Syst. 2009 May;25(5):619-26. doi: 10.1007/s00381-009-0838-2. PMID: 19301014. |
| 16. Riina HA, Fraser JF, Fralin S, Knopman J, Scheff RJ, Boockvar JA. Superselective intraarterial cerebral infusion of bevacizumab: a revival of interventional neuro-oncology for malignant glioma. J Exp Ther Oncol. 2009;8(2):145-50. PMID: 20192120. |
| 17. Miyamoto J, Tatsuzawa K, Owada K, Kawabe T, Sasajima H, Mineura K. Usefulness and limitations of fluorine-18-fluorodeoxyglucose positron emission tomography for the detection of malignancy of orbital tumors. Neurol Med Chir (Tokyo). 2008;48(11):495-9; discussion 499. PMID: 19029776. |
| 18. Yaghoubi SS, Jensen MC, Satyamurthy N, Budhiraja S, Paik D, Czernin J, Gambhir SS. Noninvasive detection of therapeutic cytolytic T cells with 18F-FHBG PET in a patient with glioma. Nat Clin Pract Oncol. 2009 Jan;6(1):53-8. doi:10.1038/ncponc1278. PMID: 19015650. |
| 19. Prins RM, Cloughesy TF, Liau LM. Cytomegalovirus immunity after vaccination with autologous glioblastoma lysate. N Engl J Med. 2008 Jul 31;359(5):539-41. doi: 10.1056/NEJMc0804818. PMID: 18669440. |
| 20. Editorial Board of Plastic and Reconstructive Surgery. Management of a complex scalp defect. Plast Reconstr Surg. 2008 Aug;122(2):623-5. doi:10.1097/PRS.0b013e31817d7f91.PMID: 18626384. |
| 21. Idbaih A, Kouwenhoven M, Jeuken J, Carpentier C, Gorlia T, Kros JM, French P, Teepen JL, Delattre O, Delattre JY, van den Bent M, Hoang-Xuan K. Chromosome 1p loss evaluation in anaplastic oligodendrogliomas. Neuropathology. 2008 Aug;28(4):440-3. doi: 10.1111/j.1440-1789.2008.00863.x. PMID: 18312547. |
| 22. Nabika S, Kiya K, Satoh H, Mizoue T, Araki H, Oshita J, Nishisaka T, Kurisu K, Sugiyama K. Primary angiitis of the central nervous system mimicking dissemination from brainstem neoplasm: a case report. Surg Neurol. 2008 Aug;70(2):182-5; discussion 185. doi: 10.1016/j.surneu.2007.05.008. PMID: 18261774. |
| 23. Dehdashti AR, Sharma S, Laperriere N, Bernstein M. Coincidence vs cause: cure in three glioblastoma patients treated with brachytherapy. Can J Neurol Sci. 2007 Aug;34(3):339-42. PMID: 17803034. |
| 24. H-Stenstam B, Pellettieri L, Sköld K, Rezaei A, Brun A. Neuropathological postmortem evaluation of BNCT for GBM. Acta Neurol Scand. 2007 Sep;116(3):169-76. PMID: 17714330. |
| 25. Sendi P, Puric E, Schönenberger A, Bargetzi M. Chronic lymphocytic leukemia and loss of strength in the right arm--not a typical combination. Praxis (Bern 1994). 2007 May 2;96(18):729-32. PMID: 17520841. |
| 26. Broniscer A, Panetta JC, O'Shaughnessy M, Fraga C, Bai F, Krasin MJ, Gajjar A, Stewart CF. Plasma and cerebrospinal fluid pharmacokinetics of erlotinib and its active metabolite OSI-420. Clin Cancer Res. 2007 Mar 1;13(5):1511-5. PMID: 17332296. |
| 27. Oshiro S, Tsugu H, Komatsu F, Ohnishi H, Ueno Y, Sakamoto S, Fukushima T, Soma G. Evaluation of intratumoral administration of tumor necrosis factor-alpha in patients with malignant glioma. Anticancer Res. 2006 Nov-Dec;26(6A):4027-32. PMID: 17195453. |
| 28. Classen CF, Warmuth-Metz M, Papke K, Trotter A, Wolff JE, Wagner S. Late response to radiochemotherapy in pediatric glioblastoma: report on two patients treated according to HIT-GBM protocols. Pediatr Hematol Oncol. 2006 Dec;23(8):631-7. PMID: 17065139. |
| 29. Koch D, Hundsberger T, Boor S, Kaina B. Local intracerebral administration of O(6)-benzylguanine combined with systemic chemotherapy with temozolomide of a patient suffering from a recurrent glioblastoma. J Neurooncol. 2007 Mar;82(1):85-9. PMID: 17031555. |
| 30. Uzuka T, Takahashi H, Tanaka R. Interstitial hyperthermia with intra-arterial injection of adriamycin for malignant glioma. Neurol Med Chir (Tokyo). 2006 Jan;46(1):19-23; discussion 23. PMID: 16434821. |
| 31. Chamoun RB, Alaraj AM, Al Kutoubi AO, Abboud MR, Haddad GF. Role of temozolomide in spinal cord low grade astrocytomas: results in two paediatric patients. Acta Neurochir (Wien). 2006 Feb;148(2):175-9; discussion 180. PMID: 16374565. |
| 32. Salomão JF, Pone MV, da Silva AR, Leibinger RD, Bellas AR, Campos JM, Garrido JR, Vanazzi E, de Barros AC, Pone SM, Boechat MB. Positive reaction for cysticercosis and multicentric anaplastic oligoastrocytoma. Childs Nerv Syst. 2006 Feb;22(2):182-5. PMID: 15778869. |
| 33. Kageji T, Nagahiro S, Mizobuchi Y, Toi H, Nakagawa Y, Kumada H. Radiation injury of boron neutron capture therapy using mixed epithermal- and thermal neutron beams in patients with malignant glioma. Appl Radiat Isot. 2004 Nov;61(5):1063-7. PMID: 15308193. |
| 34. de Wit MC, de Bruin HG, Eijkenboom W, Sillevis Smitt PA, van den Bent MJ. Immediate post-radiotherapy changes in malignant glioma can mimic tumor progression. Neurology. 2004 Aug 10;63(3):535-7. PMID: 15304589. |
| 35. ayashi Y, Hirata K, Tanaka H, Yamada T. Quinacrine administration to a patient with Creutzfeldt-Jakob disease who received a cadaveric dura mater graft--an EEG evaluation. Rinsho Shinkeigaku. 2003 Jul;43(7):403-8. Japanese. PMID: 14582366. |
| 36. Okada H, Lieberman FS, Edington HD, Witham TF, Wargo MJ, Cai Q, Elder EH, Whiteside TL, Schold SC Jr, Pollack IF. Autologous glioma cell vaccine admixed with interleukin-4 gene transfected fibroblasts in the treatment of recurrent glioblastoma: preliminary observations in a patient with a favorable response to therapy. J Neurooncol. 2003 Aug-Sep;64(1-2):13-20. PMID: 12952282. |
| 37. Colosimo C, di Lella GM, Tartaglione T, Riccardi R. Neuroimaging of thalamic tumors in children. Childs Nerv Syst. 2002 Aug;18(8):426-39. Epub 2002 Jul 30. PubMed PMID: 12192502. |
| 38. Inamura T, Ikezaki K, Hirokawa E, Kawamura T, Yoshiura T, Mihara F, Sueyasu M, Irita K, Takahashi S, Fukui M. Clinical trial of bradykinin-enhancing chemotherapy for a recurrent malignant glioma: a case report. No Shinkei Geka. 2001 Nov;29(11):1107-13. Japanese. PMID: 11758319. |
| 39. Cipri S, Mannino R, Ruggieri R, Gambardella G. Clinical evaluation of thallium-201 single photon emission computed tomography in equivocal neuroradiological supratentorial lesions. J Neurosurg Sci. 2001 Jun;45(2):75-82. PMID: 11533531. |
| 40. akabayashi T, Yoshida J, Mizuno M, Kajita Y. Intratumoral microinfusion of nimustine (ACNU) for recurrent glioma. Brain Tumor Pathol. 2001;18(1):23-8. PMID: 11517970. |
| 41. Farmer JP, Montes JL, Freeman CR, Meagher-Villemure K, Bond MC, O'Gorman AM. Brainstem Gliomas. A 10-year institutional review. Pediatr Neurosurg. 2001Apr;34(4):206-14. PMID: 11359114. |
| 42. Trouillas P, Honnorat J, Bret P, Jouvet A, Gerard JP. Redifferentiation therapy in brain tumors: long-lasting complete regression of glioblastomas and an anaplastic astrocytoma under long term 1-alpha-hydroxycholecalciferol. J Neurooncol. 2001 Jan;51(1):57-66. PMID: 11349882. |
| 43. Kallén K, Burtscher IM, Holtås S, Ryding E, Rosén I. 201Thallium SPECT and 1H-MRS compared with MRI in chemotherapy monitoring of high-grade malignant astrocytomas. J Neurooncol. 2000;46(2):173-85. PMID: 10894370. |
| 44. Tanaka S, Kamitani H, Amin MR, Watanabe T, Oka H, Fujii K, Nagashima T, Hori T. Preliminary individual adjuvant therapy for gliomas based on the results of molecular biological analyses for drug-resistance genes. J Neurooncol. 2000;46(2):157-71. PMID: 10894369. |
| 45. Bakhshandeh A, Bruns I, Eberhardt K, Wiedemann GJ. Chemotherapy in combination with whole-body hyperthermia in advanced malignant pleural mesothelioma. Dtsch Med Wochenschr. 2000 Mar 17;125(11):317-9. German. PMID: 10761473. |
| 46. Morgenstern LB, Frankowski RF. Brain tumor masquerading as stroke. J Neurooncol. 1999 Aug;44(1):47-52. PMID: 10582668. |
| 47. Cheeseman SL, Ranson MR. Cerebral metastases in malignant mesothelioma: a case report. Eur J Cancer Care (Engl). 1999 Jun;8(2):104-6. PMID:10476113. |
| 48. Ghaziuddin N, DeQuardo JR, Ghaziuddin M, King CA. Electroconvulsive treatment of a bipolar adolescent postcraniotomy for brain stem astrocytoma. J Child Adolesc Psychopharmacol. 1999;9(1):63-9. PMID: 10357519. |
| 49. Krings T, Chiappa KH, Cuffin BN, Buchbinder BR, Cosgrove GR. Accuracy of electroencephalographic dipole localization of epileptiform activities associated with focal brain lesions. Ann Neurol. 1998 Jul;44(1):76-86. PMID: 9667595. |
| 50. Calaminus G, Janssen G, Lenard HG, Bock WJ, Reifenberger G, Schmitt G, Göbel U. Combined therapy of medulloblastoma: review of 46 patients treated in a single institution. Neuropediatrics. 1998 Apr;29(2):102-7. PMID: 9638665. |
| 51. Tsuda H, Sata M, Kumabe T, Hara H, Eriguchi N, Sugita Y, Nagamatsu H. Quick response of advanced cancer to chemoradiation therapy with antineoplastons. Oncol Rep. 1998 May-Jun;5(3):597-600. PMID: 9538158. |
| 52. DiGiovanna JJ, Patronas N, Katz D, Abangan D, Kraemer KH. Xeroderma pigmentosum: spinal cord astrocytoma with 9-year survival after radiation and isotretinoin therapy. J Cutan Med Surg. 1998 Jan;2(3):153-8. PMID: 9479081. |
| 53. Weitzner MA, Meyers CA. Cognitive functioning and quality of life in malignant glioma patients: a review of the literature. Psychooncology. 1997 Sep;6(3):169-77. PMID: 9313282. |
| 54. Cloughesy TF, Woods RP, Black KL, Couldwell WT, Law RE, Hinton DR. Prolonged treatment with biologic agents for malignant glioma: a case study with high dose tamoxifen. J Neurooncol. 1997 Oct;35(1):39-45. PMID: 9266439. |
| 55. Wetmore SJ, Koike KJ, Bloomfield SM. Profound hearing loss from intravertebral artery cisplatin. Otolaryngol Head Neck Surg. 1997 Feb;116(2):234-7. PMID: 9051071. |
| 56. Kaba SE, DeMonte F, Bruner JM, Kyritsis AP, Jaeckle KA, Levin V, Yung WK. The treatment of recurrent unresectable and malignant meningiomas with interferon alpha-2B. Neurosurgery. 1997 Feb;40(2):271-5. PMID: 9007858. |
| 57. Tanaka T, Kobayashi T, Kida Y, Oyama H, Niwa M. The results of gamma knife radiosurgery for malignant skull base tumors. No Shinkei Geka. 1996 Mar;24(3):235-9. Japanese. PMID: 8851952. |
| 58. Fukahori T, Tahara T, Mihara F, Kato A, Masumoto H, Kudo S, Tsuji T, Tabuchi K. Diagnostic value of high N-isopropyl-p-[123I] iodoamphetamine (IMP) uptake in brain tumors. Nihon Igaku Hoshasen Gakkai Zasshi. 1996 Jan;56(1):53-9. Japanese. PMID: 8857100. |
| 59. Kumon Y, Zenke K, Ohta S, Hatakeyama T, Sakaki S, Yanagihara N. Operative results in fourteen cases of paranasal sinus and anterior cranial fossa lesions surgically treated by an extended transbasal approach. No Shinkei Geka. 1995 Oct;23(10):889-95. Japanese. PMID: 7477698 |
| 60. Williams PC, Henner WD, Roman-Goldstein S, Dahlborg SA, Brummett RE, Tableman M, Dana BW, Neuwelt EA. Toxicity and efficacy of carboplatin and etoposide in conjunction with disruption of the blood-brain tumor barrier in the treatment of intracranial neoplasms. Neurosurgery. 1995 Jul;37(1):17-27; discussion 27-8. PMID: 8587686. |
| 61. Nebeling LC, Miraldi F, Shurin SB, Lerner E. Effects of a ketogenic diet on tumor metabolism and nutritional status in pediatric oncology patients: two case reports. J Am Coll Nutr. 1995 Apr; 14(2):202-8. PMID: 7790697. |
| 62. Inoue HK, Hayashi S, Ishihara J, Horikoshi S, Zama A, Hirato M, Shibazaki T, Andou Y, Ohye C. Fractionated Gamma Knife radiosurgery for malignant gliomas: neurobiological effects and FDG-PET studies. Stereotact Funct Neurosurg. 1995;64 Suppl 1:249-57. PMID: 8584835. |
| 63. Ohnishi H, Koizumi K, Uchiyama G, Yamaguchi M, Okada J, Ogata H, Toyama K, Monzawa S, Oba H, Araki T, et al. Evaluation of malignancy and viability of brain tumors by 201Tl SPECT: the correlation between 201Tl SPECT and pathology, clinical progress and the intensity of enhancement on CT images. Nihon Igaku Hoshasen Gakkai Zasshi. 1994 Dec 25;54(14):1388-98. Japanese. PMID: 7596768. |
| 64. Filov VA, Gershanovich ML, Ivin BA, Danova LA, Gurchin FA, Naryshkin AG, Leshchinskiĭ VI, Zemskaia AG, Nikiforov BM, Breĭvis PV. Therapy of primary brain tumors with segidrin. Vopr Onkol. 1994;40(7-12):332-6. Russian. PMID: 7610631. |
| 65. Byrd SE, Darling CF, Wilczynski MA. Magnetic resonance imaging of the central nervous system in children with a new nonionic gadolinium contrast agent--gadoteridol injection (ProHance). J Natl Med Assoc. 1993 May;85(5):361-6. PMID: 8496990. |
| 66. Heafield MT, Carey M, Williams AC, Cullen M. Neoplastic angioendotheliomatosis: a treatable "vascular dementia" occurring in an immunosuppressed transplant patient. Clin Neuropathol. 1993 Mar-Apr;12(2):102-6. PMID: 8477550. |
| 67. Barnett GH, McKenzie RL, Ramos L, Palmer J. Nonvolumetric stereotaxy-assisted craniotomy. Results in 50 consecutive cases. Stereotact Funct Neurosurg. 1993;61(2):80-95. PMID: 8197330. |
| 68. Tanaka S, Tabuchi S, Watanabe K, Takigawa H, Akatsuka K, Numata H, Hokama Y, Hori T. Preventive effects of interleukin 1 beta for ACNU-induced myelosuppression in malignant brain tumors: the experimental and preliminary clinical studies. J Neurooncol. 1992 Oct;14(2):159-68. PMID: 1331350. |
| 69. Ghosh C, Lazarus HM, Hewlett JS, Creger RJ. Fluctuation of serum phenytoin concentrations during autologous bone marrow transplant for primary central nervous system tumors. J Neurooncol. 1992 Jan;12(1):25-32. PMID: 1311752. |
| 70. Sano K, Kawasaki C, Sato K, Iwasaki A, Ishizuka E. A case of renal cell carcinoma similar to rhabdoid tumor of kidney in an adult; trial of radiation and chemotherapy. Hinyokika Kiyo. 1991 Dec;37(12):1699-702. Japanese. PMID: 1785395. |
| 71. Kikuchi K, Mineura K, Sakamoto T, Kowada M, Sageshima M. Efficacy of preoperative irradiation in midline oligodendrogliomas. Report of two cases. Neurol Med Chir (Tokyo). 1991 Dec;31(13):912-8. Japanese. PMID: 1726252. |
| 72. Guthkelch AN, Carter LP, Cassady JR, Hynynen KH, Iacono RP, Johnson PC, Obbens EA, Roemer RB, Seeger JF, Shimm DS, et al. Treatment of malignant brain tumors with focused ultrasound hyperthermia and radiation: results of a phase I trial. J Neurooncol. 1991 Jun;10(3):271-84. PMID: 1654406. |
| 73. Kobayashi T, Kida Y, Matsui M, Amemiya Y. Interstitial hyperthermia of malignant brain tumors using implant heating system (IHS). No Shinkei Geka. 1990 Mar;18(3):247-52. Japanese. PMID: 2359474. |
| 74. Vander JF, Kincaid MC, Hegarty TJ, Page M, Averill D, Junck L, Greenberg HS. The ocular effects of intracarotid bromodeoxyuridine and radiation therapy in the treatment of malignant glioma. Ophthalmology. 1990 Mar;97(3):352-7. PMID: 2186331. |
| 75. Kawano H, Hayashi M, Satoh K, Ishii H, Nakatsugawa S, Ishii Y. A trial of ACNU and radiation therapy with sensitizing agents for malignant gliomas. No To Shinkei. 1989 Nov;41(11):1071-5. Japanese. PMID: 2620008. |
| 76. Shimizu K, Tamura K, Yamada M, Okamoto Y, Miyao Y, Park K, Matsui Y, Hayakawa T, Takimoto H, Mogami H. Adoptive immunotherapy in patients with medulloblastoma by LAK cells. No To Shinkei. 1989 Oct;41(10):991-5. Japanese. PMID: 2605046. |
| 77. Vaquero J, Martínez R, Oya S, Coca S, Barbolla L, Ramiro J, Salazar FG. Intratumoural injection of autologous lymphocytes plus human lymphoblastoid interferon for the treatment of glioblastoma. Acta Neurochir (Wien). 1989;98(1-2):35-41. PMID: 2472737. |
| 78. Ingram M, Shelden CH, Jacques S, Skillen RG, Bradley WG, Techy GB, Freshwater DB, Abts RM, Rand RW. Preliminary clinical trial of immunotherapy for malignant glioma. J Biol Response Mod. 1987 Oct;6(5):489-98. PMID: 3316510. |
| 79. Kanazawa I, Shiraishi K, Kamitani H, Sato J, Masuzawa H. Intraoperative ultrasonography through a burr hole: clinical trial of ultrasound-guided stereotactic surgery. No Shinkei Geka. 1986 Mar;14(3 Suppl):295-300. Japanese. PMID: 3010152. |
| 80. Allen JC. Childhood brain tumors: current status of clinical trials in newly diagnosed and recurrent disease. Pediatr Clin North Am. 1985 Jun;32(3):633-51. PMID: 3889800. |
| 81. Cooper JS. Radiotherapy of malignant melanoma. Dermatol Clin. 1985 Apr;3(2):341-50. PMID: 3830495. |
| 82. Moritake K, Handa H, Yamashita J, Takeuchi J, Taki W, Takebe Y, Konishi T. STA-MCA anastomosis in patients with skull base tumours involving the internal carotid artery--haemodynamic assessment by ultrasonic Doppler flowmeter. Acta Neurochir (Wien). 1984;72(1-2):95-110. PMID: 6741650. |
| 83. Nagai M, Arai T. Clinical effect of interferon in malignant brain tumours. Neurosurg Rev. 1984;7(1):55-64. PMID: 6379511. |
| 84. Yamashita J, Handa H, Tokuriki Y, Ha YS, Otsuka SI, Suda K, Taki W. Intra-arterial ACNU therapy for malignant brain tumors. Experimental studies and preliminary clinical results. J Neurosurg. 1983 Sep;59(3):424-30. PMID: 6577143. |
| 85. Nakazawa S, Itoh Y, Shimura T, Matsumoto M, Yajima K. New management of brain neoplasms. 2. Local injection of adriamycin. No Shinkei Geka. 1983 Aug;11(8):821-7. Japanese. PubMed PMID: 6314166. |
| 86. Salcman M, Samaras GM. Interstitial microwave hyperthermia for brain tumors. Results of a phase-1 clinical trial. J Neurooncol. 1983;1(3):225-36. PMID: 6088715. |
| 87. Hill SA, Neuwelt EA. Intra-arterial chemotherapy following blood-brain barrier disruption in a patient with malignant glioma. J Neurosurg Nurs. 1982 Apr;14(2):94-7. PMID: 6811704. |
| 88. Nakazawa S, Ohwaki K, Shimura T, Itoh Y, Yajima K. A new treatment of malignant brain tumor -1: local injection of bleomycin (author's transl). No Shinkei Geka. 1981 Dec;9(13):1487-93. Japanese. PMID: 6179006. |
| 89. Allen JC, Helson L. High-dose cyclophosphamide chemotherapy for recurrent CNS tumors in children. J Neurosurg. 1981 Nov;55(5):749-56. PubMed PMID: 7310496. |
| 90. Greenberg HS, Ensminger WD, Seeger JF, Kindt GW, Chandler F, Doan K, Dakhil SR. Intra-arterial BCNU chemotherapy for the treatment of malignant gliomas of the central nervous system: a preliminary report. Cancer Treat Rep. 1981 Sep-Oct;65(9-10):803-10. PMID: 7023671. |
| 91. Ransohoff J, Martin BF, Medrek TJ, Harris MN, Golomb FM, Wright JC. Preliminary clinical study of mithramycin (nsc-24559) in primary tumors of the central nervous system. Cancer Chemother Rep. 1965 Dec;49:51-7. PMID: 4285360. |

**Table 5 List 21 DC protocol studies and comments for being no clinical data**

| **Excluding 21 DC protocol studies and comments for no clinical data** |
| --- |
| 1. Candolfi M, Yagiz K, Foulad D, Alzadeh GE, Tesarfreund M, Muhammad AK, Puntel M, Kroeger KM, Liu C, Lee S, Curtin JF, King GD, Lerner J, Sato K, Mineharu Y, Xiong W, Lowenstein PR, Castro MG. Release of HMGB1 in response to proapoptotic glioma killing strategies: efficacy and neurotoxicity. Clin Cancer Res. 2009 Jul 1;15(13):4401-14. doi: 10.1158/1078-0432.CCR-09-0155.PMID: 19570774. |
| 2. La Ferla-Brühl K, Westhoff MA, Karl S, Kasperczyk H, Zwacka RM, Debatin KM, Fulda S. NF-kappaB-independent sensitization of glioblastoma cells for TRAIL-induced apoptosis by proteasome inhibition. Oncogene. 2007 Jan 25;26(4):571-82. PMID: 16909119. |
| 3. Schultze K, Böck B, Eckert A, Oevermann L, Ramacher D, Wiestler O, Roth W. Troglitazone sensitizes tumor cells to TRAIL-induced apoptosis via down-regulation of FLIP and Survivin. Apoptosis. 2006 Sep;11(9):1503-12. PMID: 16820965. |
| 4. Kuijlen JM, Mooij JJ, Platteel I, Hoving EW, van der Graaf WT, Span MM, Hollema H, den Dunnen WF. TRAIL-receptor expression is an independent prognostic factor for survival in patients with a primary glioblastoma multiforme. J Neurooncol. 2006 Jun;78(2):161-71. PMID: 16544055. |
| 5. Uzzaman M, Benveniste RJ, Keller G, Germano IM. Embryonic stem cell-derived astrocytes: a novel gene therapy vector for brain tumors. Neurosurg Focus. 2005 Sep 15;19(3):E6. PMID: 16190605. |
| 6. Ashley DM, Riffkin CD, Muscat AM, Knight MJ, Kaye AH, Novak U, Hawkins CJ. Caspase 8 is absent or low in many ex vivo gliomas. Cancer. 2005 Oct 1;104(7):1487-96. PMID: 16080161. |
| 7. Braeuninger S, Chamaon K, Kropf S, Mawrin C, Wiedemann FR, Hartig R, Schoeler S, Dietzmann K, Kirches E. Short incubation with 2-methoxyestradiol kills malignant glioma cells independent of death receptor 5 upregulation. Clin Neuropathol. 2005 Jul-Aug;24(4):175-83. PMID: 16033134. |
| 8. Shah K, Bureau E, Kim DE, Yang K, Tang Y, Weissleder R, Breakefield XO. Glioma therapy and real-time imaging of neural precursor cell migration and tumor regression. Ann Neurol. 2005 Jan;57(1):34-41. PMID: 15622535. |
| 9. Choi C, Jeong E, Benveniste EN. Caspase-1 mediates Fas-induced apoptosis and is up-regulated by interferon-gamma in human astrocytoma cells. J Neurooncol. 2004 Mar-Apr;67(1-2):167-76. PMID: 15072464. |
| 10. Song JH, Song DK, Pyrzynska B, Petruk KC, Van Meir EG, Hao C. TRAIL triggers apoptosis in human malignant glioma cells through extrinsic and intrinsic pathways. Brain Pathol. 2003 Oct;13(4):539-53. PMID: 14655759. |
| 11. Roa WH, Chen H, Fulton D, Gulavita S, Shaw A, Th'ng J, Farr-Jones M, Moore R, Petruk K. X-linked inhibitor regulating TRAIL-induced apoptosis in chemoresistant human primary glioblastoma cells. Clin Invest Med. 2003 Oct;26(5):231-42. PMID: 14596484. |
| 12. James CD, Cavenee WK. Stem cells for treating glioblastoma: how close to reality? Neuro Oncol. 2009 Apr;11(2):101. doi: 10.1215/15228517-2009-016. PMID: 19237733. |
| 13. Verbrugge I, Johnstone RW. Regulating the TRAIL of destruction: how A20 protects glioblastomas from TRAIL-mediated death. Cancer Discov. 2012 Feb;2(2):112-4.  doi: 10.1158/2159-8290.CD-11-0350.PMID: 22585854. |
| 14. James CD, Cavenee WK. Stem cells for treating glioblastoma: how close to reality? Neuro Oncol. 2009 Apr;11(2):101. doi: 10.1215/15228517-2009-016. PMID: 19237733 |
| 15. Hollander AB, Dorsey JF. FLIPing the apoptosis switch on in brain tumors using Troglitazone and TRAIL. Cancer Biol Ther. 2008 Dec;7(12):1991-3. PMID: 19158481. |
| 16. Kyritsis AP, Tachmazoglou F, Rao JS, Puduvalli VK. Bortezomib sensitizes human astrocytoma cells to tumor necrosis factor related apoptosis-inducing ligand induced apoptosis. Clin Cancer Res. 2007 Nov 1;13(21):6540-1. PMID: 17975168. |
| 17. Kyritsis AP, Rao JS, Puduvalli VK. Radio-responsive TRAIL gene therapy for malignant gliomas. Cancer Gene Ther. 2007 Dec;14(12):1002. PMID: 17704752. |
| 18. Stupp R, Tonn JC, Brada M, Pentheroudakis G; ESMO Guidelines Working Group. High-grade malignant glioma: ESMO Clinical Practice Guidelines for diagnosis, treatment and follow-up. Ann Oncol. 2010 May;21 Suppl 5:v190-3. PMID: 20555079. |
| 19. Fadul CE, Wen PY, Kim L, Olson JJ. Cytotoxic chemotherapeutic management of newly diagnosed glioblastoma multiforme. J Neurooncol. 2008 Sep;89(3):339-57. doi: 10.1007/s11060-008-9615-4. PMID: 18712284. |
| 20. Buatti J, Ryken TC, Smith MC, Sneed P, Suh JH, Mehta M, Olson JJ. Radiation therapy of pathologically confirmed newly diagnosed glioblastoma in adults. J Neurooncol. 2008 Sep;89(3):313-37. doi: 10.1007/s11060-008-9617-2. PMID: 18712283. |
| 21. Brady LW. A new treatment for high grade gliomas of the brain. Bull Mem Acad R Med Belg. 1998;153(5-6):255-61,discussion 261-2. PMID: 9988932. |

**Table 6 List 22 clinical trials for no appropriate control arm**

| **Excluding 22 clinical trials for no appropriate control arm** |
| --- |
| 1.Ardon H, De Vleeschouwer S, Van Calenbergh F, Claes L, Kramm CM, Rutkowski S, Wolff JE, Van Gool SW. Adjuvant dendritic cell-based tumour vaccination for children with malignant brain tumours. Pediatr Blood Cancer. 2010 Apr;54(4):519-25.  doi: 10.1002/pbc.22319. PMID: 19852061.  **Participants：**Malignant brain tumour patients  **Interventions：**DC vaccination  **Comparison：**Which brain tumor immunotherapy more effective? High grade glioma (HGG) or medulloblastoma (MB)/primitive neuro-ectodermal tumour (PNET) or ependymoma and atypical teratoid-rhabdoid tumour (ATRT)  **Outcomes：**Progression-free survival (PFS) and overall survival (OS)  **Setting：**Only immunotherapy group |
| 2. Sampson JH, Archer GE, Mitchell DA, Heimberger AB, Herndon JE 2nd, Lally-Goss D, McGehee-Norman S, Paolino A, Reardon DA, Friedman AH, Friedman HS, Bigner DD. An epidermal growth factor receptor variant III-targeted vaccine is safe and immunogenic in patients with glioblastoma multiforme. Mol Cancer Ther. 2009 Oct;8(10):2773-9. doi: 10.1158/1535-7163.MCT-09-0124. PMID: 19825799.  **Participants：**Glioblastoma multiforme (GBM) patients  **Interventions：**An epidermal growth factor receptor variant III–targeted DC vaccination  **Comparison：**Epidermal growth factor receptor variant III-targeted vaccine treatment glioblastoma patient safety and immunogenicity  **Outcomes：**Time to progression (TTP) and survival  **Setting：**Only immunotherapy group |
| 3. Prins RM, Soto H, Konkankit V, Odesa SK, Eskin A, Yong WH, Nelson SF, Liau LM. Gene expression profile correlates with T-cell infiltration and relative survival in glioblastoma patients vaccinated with dendritic cell immunotherapy. Clin Cancer Res. 2011 Mar 15;17(6):1603-15. doi: 10.1158/1078-0432.CCR-10-2563. PMID: 21135147.  **Participants：**Newly diagnosed and recurrent glioblastoma patients  **Interventions：**Autologous tumor lysate–pulsed DC vaccination in conjunction with toll-like receptor(TLR) agonists  **Comparison：**To assess the feasibility, safety, and toxicity of autologous tumor lysate–pulsed dendritic cell (DC) vaccination and toll-like receptor (TLR) agonists in patients with newly diagnosed and recurrent glioblastoma.  **Outcomes：**Time to progression (TTP) and survival  **Setting：**Only immunotherapy group |
| 4. Fadul CE, Fisher JL, Hampton TH, Lallana EC, Li Z, Gui J, Szczepiorkowski ZM, Tosteson TD, Rhodes CH, Wishart HA, Lewis LD, Ernstoff MS. Immune response in patients with newly diagnosed glioblastoma multiforme treated with intranodal autologous tumor lysate-dendritic cell vaccination after radiation chemotherapy. J Immunother. 2011 May;34(4):382-9. doi: 10.1097/CJI.0b013e318215e300. PMID: 21499132.  **Participants：**Newly diagnosed GBM patients  **Interventions：**Autologous tumor lysate-dendritic cell vaccination and radiation chemotherapy  **Comparison：**To test the hypothesis that diminishing the activity of regulatory T cells (TREG) and improving immune cell access to the central nervous system would enhance response to immune therapy and consequently affect survival of patients with GBM.  **Outcomes：**Progression-free survival (PFS) and Overall survival (OS)  **Setting：**Only immunotherapy group |
| 5. Ardon H, Van Gool S, Lopes IS, Maes W, Sciot R, Wilms G, Demaerel P, Bijttebier P, Claes L, Goffin J, Van Calenbergh F, De Vleeschouwer S. Integration of autologous dendritic cell-based immunotherapy in the primary treatment for patients with newly diagnosed glioblastoma multiforme: a pilot study. J Neurooncol. 2010 Sep;99(2):261-72.  doi: 10.1007/s11060-010-0131-y. PMID: 20146084.  **Participants：**Newly diagnosed GBM patients  **Interventions：**Autologous tumor lysate-dendritic cell vaccination and radiation chemotherapy  **Comparison：**To assess clinical and immunological feasibility and toxicity in patients with GBM.  **Outcomes：**PFS and OS  **Setting：**Only immunotherapy group |
| 6. Ardon H, Van Gool SW, Verschuere T, Maes W, Fieuws S, Sciot R, Wilms G, Demaerel P, Goffin J, Van Calenbergh F, Menten J, Clement P, Debiec-Rychter M, De Vleeschouwer S. Integration of autologous dendritic cell-based immunotherapy in the standard of care treatment for patients with newly diagnosed glioblastoma: results of the HGG-2006 phase I/II trial. Cancer Immunol Immunother. 2012 Nov;61(11):2033-44. doi: 10.1007/s00262-012-1261-1. PMID: 22527250.  **Participants：**Newly diagnosed GBM patients  **Interventions：**Autologous tumor lysate-dendritic cell vaccination  **Comparison：**In the trial feasibility, toxicity, and clinical efficacy of the full integration of DC-based tumor vaccination into standard postoperative radiochemotherapy are studied in patient with newly diagnosed glioblastoma  **Outcomes：**PFS and OS  **Setting：**Only immunotherapy group |
| 7. De Vleeschouwer S, Fieuws S, Rutkowski S, Van Calenbergh F, Van Loon J, Goffin J, Sciot R, Wilms G, Demaerel P, Warmuth-Metz M, Soerensen N, Wolff JE, Wagner S, Kaempgen E, Van Gool SW. Postoperative adjuvant dendritic cell-based immunotherapy in patients with relapsed glioblastoma multiforme. Clin Cancer Res. 2008 May 15;14(10):3098-104.  doi: 10.1158/1078-0432.CCR-07-4875. PMID: 18483377.  **Participants：**Relapsed glioblastoma multiforme patients  **Interventions：**Autologous tumor lysate-dendritic cell vaccination and radiation chemotherapy  **Comparison：**To investigate the therapeutic role of adjuvant vaccination with autologous mature dendritic cells (DC) loaded with tumor lysates derived from autologous, resected glioblastoma multiforme (GBM) at time of relapse.  **Outcomes：**KPS, PFS and OS  **Setting：**Only immunotherapy group |
| 8. Walker DG, Laherty R, Tomlinson FH, Chuah T, Schmidt C. Results of a phase I dendritic cell vaccine trial for malignant astrocytoma: potential interaction with adjuvant chemotherapy. J Clin Neurosci. 2008 Feb;15(2):114-21. PMID: 18083572.  **Participants：**Malignant astrocytoma patients  **Interventions：**Dendritic cell vaccination and chemotherapy  **Comparison：**Is the treatment safe, induces an immune response and appears to combine with adjuvant chemotherapy to produce favourable tumour responses?  **Outcomes：**OS  **Setting：**Only immunotherapy group |
| 9. Yu JS, Wheeler CJ, Zeltzer PM, Ying H, Finger DN, Lee PK, Yong WH, Incardona F, Thompson RC, Riedinger MS, Zhang W, Prins RM, Black KL. Vaccination of malignant glioma patients with peptide-pulsed dendritic cells elicits systemic cytotoxicity and intracranial T-cell infiltration. Cancer Res. 2001 Feb 1;61(3):842-7. PMID: 11221866.  **Participants：**Malignant glioma patients  **Interventions：**Autologous peptide-pulsed dendritic cell vaccine  **Comparison：**This phase I study demonstrated the feasibility, safety, and bioactivity of an autologous peptide-pulsed dendritic cell vaccine for patients with malignant glioma.  **Outcomes：**OS and time to progression  **Setting：**Only immunotherapy group |
| 10. Yamanaka R, Abe T, Yajima N, Tsuchiya N, Homma J, Kobayashi T, Narita M, Takahashi M, Tanaka R. Vaccination of recurrent glioma patients with tumour lysate-pulsed dendritic cells elicits immune responses: results of a clinical phase I/II trial. Br J Cancer. 2003 Oct 6;89(7):1172-9. PMID: 14520441.  **Participants：**Recurrent glioma patients  **Interventions：**Tumour lysate-pulsed dendritic cells  **Comparison：**This study demonstrated the safety and antitumour effects of autologous tumour lysate-pulsed dendritic cell therapy for patients with malignant glioma.  **Outcomes：**OS and adverse effects  **Setting：**Only immunotherapy group |
| 11. De Vleeschouwer S, Ardon H, Van Calenbergh F, Sciot R, Wilms G, van Loon J, Goffin J, Van Gool S. Stratification according to HGG-IMMUNO RPA model predicts outcome in a large group of patients with relapsed malignant glioma treated by adjuvant postoperative dendritic cell vaccination. Cancer Immunol Immunother. 2012 Nov;61(11):2105-12.  doi: 10.1007/s00262-012-1271-z. PMID: 22565485.  **Participants：**Relapsed malignant glioma patients  **Interventions：**Postoperative dendritic cell (DC) vaccination  **Comparison：**It stratified patients with relapsed high grade glioma treated with re-operation and postoperative dendritic cell (DC) vaccination according to a simple recursive partitioning analysis (RPA) model to predict outcome.  **Outcomes：**OS  **Setting：**Only immunotherapy group |
| 12. Iwami K, Shimato S, Ohno M, Okada H, Nakahara N, Sato Y, Yoshida J, Suzuki S, Nishikawa H, Shiku H, Natsume A, Wakabayashi T. Peptide-pulsed dendritic cell vaccination targeting interleukin-13 receptor α2 chain in recurrent malignant glioma patients with HLA-A*24/A*02 allele. Cytotherapy. 2012 Jul;14(6):733-42. doi: 10.3109/14653249.2012.666633. PMID: 22424217.  **Participants：**Recurrent malignant glioma patients  **Interventions：**Peptide-pulsed dendritic cell vaccination  **Comparison：**The objective was to evaluate the safety and clinical and immunologic responses.  **Outcomes：**OS, clinical response and immunologic assays  **Setting：**Only immunotherapy group |
| 13. Lasky JL 3rd, Panosyan EH, Plant A, Davidson T, Yong WH, Prins RM, Liau LM, Moore TB. Autologous tumor lysate-pulsed dendritic cell immunotherapy for pediatric patients with newly diagnosed or recurrent high-grade gliomas. Anticancer Res. 2013 May;33(5):2047-56. PMID: 23645755.  **Participants：**Newly diagnosed or recurrent high-grade gliomas patients  **Interventions：**Autologous tumor lysate-pulsed dendritic cell  **Comparison：**This report supports the tolerability and feasibility of tumor lysate-pulsed DC immunotherapy for pediatric glioma tumors  **Outcomes：**OS  **Setting：**Only immunotherapy group |
| 14. Okada H, Lieberman FS, Walter KA, Lunsford LD, Kondziolka DS, Bejjani GK, Hamilton RL, Torres-Trejo A, Kalinski P, Cai Q, Mabold JL, Edington HD, Butterfield LH, Whiteside TL, Potter DM, Schold SC Jr, Pollack IF. Autologous glioma cell vaccine admixed with interleukin-4 gene transfected fibroblasts in the treatment of patients with malignant gliomas. J Transl Med. 2007 Dec 19;5:67. PMID: 18093335.  **Participants：**Malignant gliomas patients  **Interventions：**Autologous glioma cell vaccine admixed with interleukin-4 gene transfected fibroblasts  **Comparison：**This study addressed the safety, feasibility and preliminary clinical activity of the vaccinations using autologous glioma cells and interleukin (IL)-4 gene transfected fibroblasts.  **Outcomes：**OS, adverse events and TTP  **Setting：**Only immunotherapy group |
| 15. Rutkowski S, De Vleeschouwer S, Kaempgen E, Wolff JE, Kühl J, Demaerel P, Warmuth-Metz M, Flamen P, Van Calenbergh F, Plets C, Sörensen N, Opitz A, Van Gool SW. Surgery and adjuvant dendritic cell-based tumour vaccination for patients with relapsed malignant glioma, a feasibility study. Br J Cancer. 2004 Nov 1;91(9):1656-62. PMID: 15477864.  **Participants：**Relapsed malignant glioma patients  **Interventions：**Dendritic cell-based tumour vaccination  **Comparison：**Tumour vaccination for patients with relapsed malignant glioma is feasible and likely beneficial for patients with minimal residual tumour burden.  **Outcomes：**OS, adverse events  **Setting：**Only immunotherapy group |
| 16. Kikuchi T, Akasaki Y, Abe T, Fukuda T, Saotome H, Ryan JL, Kufe DW, Ohno T. Vaccination of glioma patients with fusions of dendritic and glioma cells and recombinant human interleukin 12. J Immunother. 2004 Nov-Dec;27(6):452-9. PMID: 15534489.  **Participants：**Glioma patients  **Interventions：**Dendritic and glioma cells combined with recombinant human interleukin 12 (rhIL-12)  **Comparison：**The authors investigated the safety and clinical response to immunotherapy using fusions of dendritic and glioma cells combined with recombinant human interleukin 12 (rhIL-12) for the treatment of malignant glioma.  **Outcomes：**Clinical response, adverse events  **Setting：**Only immunotherapy group |
| 17. Akiyama Y, Oshita C, Kume A, Iizuka A, Miyata H, Komiyama M, Ashizawa T, Yagoto M, Abe Y, Mitsuya K, Watanabe R, Sugino T, Yamaguchi K, Nakasu Y. α-type-1 polarized dendritic cell-based vaccination in recurrent high-grade glioma: a phase I clinical trial. BMC Cancer. 2012 Dec 27;12:623. doi: 10.1186/1471-2407-12-623.PMID: 23270484.  **Participants：**Recurrent high-grade glioma patients  **Interventions：**α-type-1 polarized dendritic cell-based vaccination  **Comparison：**It investigated the effect of activated dendritic cell (DC) (α-type-1 polarized DC)-based immunotherapy on high-grade glioma patients with the HLA-A2 or A24 genotype.  **Outcomes：**Clinical response, adverse events  **Setting：**Only immunotherapy group |
| 18. Bu N, Wu H, Sun B, Zhang G, Zhan S, Zhang R, Zhou L. Exosome-loaded dendritic cells elicit tumor-specific CD8^+^ cytotoxic T cells in patients with glioma. J Neurooncol. 2011 Sep;104(3):659-67. Epub 2011 Feb 19. doi: 10.1007/s11060-011-0537-1. PMID: 21336773.  **Participants：**Glioma patients  **Interventions：**Dendritic cells  **Comparison：**It demonstrate that tumorderived exosome-loaded dendritic cells can elicit a specific CD8^+^ cytotoxic T-lymphocyte (CTL) response against autologous tumor cells in patients with malignant glioma  **Outcomes：**Clinical response  **Setting：**Only immunotherapy group |
| 19. Akasaki Y, Kikuchi T, Irie M, Yamamoto Y, Arai T, Tanaka T, Joki T, Abe T. Cotransfection of Poly(I: C) and siRNA of IL-10 into fusions of dendritic and glioma cells enhances antitumor T helper type 1 induction in patients with glioma. J Immunother. 2011 Mar;34(2):121-8. doi: 10.1097/CJI.0b013e3181e5c278. PMID: 21304408.  **Participants：**Glioma patients  **Interventions：**Dendritic cells  **Comparison：**It were studied the antitumor immune responses conferred by fusions of DCs and glioma cells.  **Outcomes：**Clinical response  **Setting：**Only immunotherapy group |
| 20. Prins RM, Wang X, Soto H, Young E, Lisiero DN, Fong B, Everson R, Yong WH, Lai A, Li G, Cloughesy TF, Liau LM. Comparison of glioma-associated antigen peptide-loaded versus autologous tumor lysate-loaded dendritic cell vaccination in malignant glioma patients. J Immunother. 2013 Feb;36(2):152-7. doi: 10.1097/CJI.0b013e3182811ae4. PMID: 23377664.  **Participants：**Malignant glioma patients  **Interventions：**Dendritic cells  **Comparison：**The objective of this study was to compare the safety, feasibility, and immune responses of malignant glioma patients on 2 different DC vaccination protocols  **Outcomes：**Clinical response  **Setting：**Only immunotherapy group |
| 21. Wheeler CJ, Black KL, Liu G, Mazer M, Zhang XX, Pepkowitz S, Goldfinger D, Ng H, Irvin D, Yu JS. Vaccination elicits correlated immune and clinical responses in glioblastoma multiforme patients. Cancer Res. 2008 Jul 15;68(14):5955-64. doi: 10.1158/0008-5472.CAN-07-5973.PMID: 18632651.  **Participants：**Glioblastoma multiforme patients  **Interventions：**Autologous tumor lysate-pulsed dendritic cell  **Comparison：**Vaccination elicits correlated immune and clinical responses in glioblastoma multiforme patients  **Outcomes：**Tumor progression (TTP) and survival (TTS)  **Setting：**Only immunotherapy group |
| 22 Bu Xing-yao, Chai Chang, Zhang Jian-guo, Li Chao-yue, Zhou Wei, Zhao Yue-wu, Zhang Yong-fu. Individual comprehensive therpy study for malignant human brain gliomas. Bull Med Res. 2004 Dec;33(12):44-46  **Participants：**Glioma patients  **Interventions：**Dendritic cell and radiotherapy  **Comparison：**To investigate the effectiveness of individual comprehensive therapy for human malignant glioma.  **Outcomes：**OS  **Setting：**Only immunotherapy group |

**Table 7 List 9 including clinical trials**

| **9 clinical trials for meta-analysis** |  |  |
| --- | --- | --- |
| 1. Chang CN, Huang YC, Yang DM, Kikuta K, Wei KJ, Kubota T, Yang WK. A phase I/II clinical trial investigating the adverse and therapeutic effects of a postoperative autologous dendritic cell tumor vaccine in patients with malignant glioma. J Clin Neurosci. 2011 Aug;18(8):1048-54. doi: 10.1016/j.jocn.2010.11.034. PMID: 21715171.  **Participants：**Glioblastoma multiforme (GBM) patients  **Interventions：**A postoperative autologous dendritic cell tumor vaccine  **Comparison：**By comparing the immunotherapy group and no-immunotherapy group to investigate the adverse and therapeutic effects of a postoperative autologous dendritic cell tumor vaccine in patients with malignant glioma  **Outcomes：**OS, adverse effect and KPS  **Setting：**Historical control   \| **Funding sources:**  This study was supported by Taiwan Department of Health-NSC Frontier Science grants DOH-89-TD.1001/DOH-92-  TD.1050 (W.K.Y.), Academia Sinica Genomic Medicine grant AS91-IBMS3PP(W.K.Y.) and CGMH research grant  No. CMRPG32017 (C.N.C.). \| \| --- \|   **Clinical data source:**  **Tumor characteristics WHO grade:** New or recurrent Ⅲ/Ⅳ  **Previous treatment:** CT/RT  **DC Arm Injection:** DCs loaded with AIT (s.c)  **Culture of DC cells:** GM-CSF, IL-4  The data were extracted from the material and methods of Chen-Nen Chang et al.’s paper.  **Clinical trial phase:** Ⅰ/Ⅱ  The data were extracted from the title of Chen-Nen Chang et al.’s paper.  **Patients (male) and control:** 17(8)/63(UK)  **Median age:** 44.7/UK  **Pre-Therapy KPS:** Median 90  **DC regimens:** 1.0-6.1×10^7^/course  The above data were extracted from Table 1 of Chen-Nen Chang et al.’s paper.  **OS%(Control): KPS**  **0.5 year:** 94.1（81） **Pre-DC treatment Post-DC treatment**  **1 year:** 58.8 （56） 82.9±8.5 77.6±9.7  **1.5 year:** 52.9 （22）  **2 year:** 41.2 （12）  **3 year:** 29.4（0）  **4 year:** 17.6（0）  **Above 5 years:** 17.6（0）  The above data were extracted from Figure 3 and Table 1 of Chen-Nen Chang et al.’s paper. | Number of patients  (control） | OS%(Control) |
| Chen-Nen Chang et al. 2011 was listed in our papers as reference 17. |  |  |

| **9 clinical trials for meta-analysis** |  |  |
| --- | --- | --- |
| 2. Yamanaka R, Homma J, Yajima N, Tsuchiya N, Sano M, Kobayashi T, Yoshida S, Abe T, Narita M, Takahashi M, Tanaka R. Clinical evaluation of dendritic cell vaccination for patients with recurrent glioma: results of a clinical phase I/II trial. Clin Cancer Res. 2005 Jun 1;11(11):4160-7. PMID: 15930352.  **Participants：**Recurrent glioma patients  **Interventions：**Dendritic cell vaccination  **Comparison：**By comparing the immunotherapy group and no-immunotherapy group to investigate the safety and the immunologic and clinical responses of dendritic cell therapy for patients with recurrent malignant glioma  **Outcomes：**OS, adverse effect  **Setting：**Historical control   \| **Funding sources:** No disclosure. \| \| --- \|   **Clinical data source:**  **Tumor characteristics WHO grade:** Recurrent Ⅲ/Ⅳ  **Previous treatment:** SR/RT/CT  **DC Arm Injection:** DCs loaded with ATL (i.d or i.t)  **Culture of DC cells:** GM-CSF, IL-4, KLH  The above data were extracted from Material and methods of Yamanaka R’s paper.  **Clinical trial phase:** Ⅰ/Ⅱ  The data were extracted from the title of Yamanaka R’s paper.  **Patients (male) and control:** 24(16)/27(UK)  **Median age:** 48.9/55.9  **Pre-Therapy KPS:** Median 62.5  The above data were extracted from Table 1 of Yamanaka R’s paper.  **DC regimens:** 3.9-240.9×10^6^/course  The above data were extracted from Table 2 of Yamanaka R’s paper.  **OS%(Control):**  **0.5 year:** 95(96)  **1 year:** 88(77)  **1.5 year:** 41(11)  **2 year:** 28(4)  **3 year:** 20(0)  **4 year:** 20(0)  **Above 5 years:** -  The above data were extracted from Figure 4 of Yamanaka R’s paper. | Number of patients  (control） | OS%(Control) |
| Yamanaka R et al. 2011 was listed in our papers as reference 18. |  |  |

| **9 clinical trials for meta-analysis** |  |  |
| --- | --- | --- |
| 3 Wheeler CJ, Das A, Liu G, Yu JS, Black KL. Clinical responsiveness of glioblastoma multiforme to chemotherapy after vaccination. Clin Cancer Res. 2004 Aug 15;10(16):5316-26. PMID: 15328167.  **Participants：**Glioblastoma multiforme patients  **Interventions：**Vaccination and chemotherapy  **Comparison：**By comparing the immunotherapy group and no-immunotherapy group to broaden the search for vaccine induced benefits  **Outcomes：**Survival and progression times  **Setting：**nonrandomized controlled trial   \| **Funding sources:** No disclosure. \| \| --- \|   **Clinical data source:**  **Tumor characteristics WHO grade:** De novo IV  **Clinical trial phase:** Ⅰ/Ⅱ  **Previous treatment:** SR/RT/CT  **DC Arm Injection:** DCs loaded with ATL or HLP  The data were extracted from the Material and methods, Results and Table 2 of Wheeler CJ’s paper.  **Patients (male) and control:** 25(11)/25(13)  **Median age:** 55/50  The above data were extracted from Table 1 of Wheeler CJ’s paper.  **DC regimens:** 10-40 ×10^6^/course  **Pre-Therapy KPS:** >60  The above data were extracted from Table 2 of Wheeler CJ’s paper.  **Culture of DC cells:** Unknown  **OS%(Control):**  **0.5 year:** 100(100)  **1 year:** 85(82)  **1.5 year:** 57(53)  **2 year:** 51(29)  **3 year:** 29(8)  **4 year:** 29(0)  **Above 5 years:** -  The above data were extracted from Figure 4 of Wheeler CJ’s paper. | Number of patients  (control） | OS%(Control) |
| Wheeler CJ et al. 2004 was listed in our papers as reference 19. |  |  |

| **9 clinical trials for meta-analysis** |  |  |
| --- | --- | --- |
| 4. Liau LM, Prins RM, Kiertscher SM, Odesa SK, Kremen TJ, Giovannone AJ, Lin JW, Chute DJ, Mischel PS, Cloughesy TF, Roth MD. Dendritic cell vaccination in glioblastoma patients induces systemic and intracranial T-cell responses modulated by the local central nervous system tumor microenvironment. Clin Cancer Res. 2005 Aug 1;11(15):5515-25. PMID: 16061868.  **Participants：**Glioblastoma patients  **Interventions：**Dendritic cell vaccination  **Comparison：**By comparing the immunotherapy group and no-immunotherapy group to evaluate this strategy for its feasibility, safety, and induction of systemic and intracranial T-cell responses in patients with glioblastoma multiforme.  **Outcomes：**Survival and adverse events  **Setting：**Historical control   \| **Funding sources:**  NIH grants CA82666(L.M.Liau), CA91545(L.M. Liau), T32-CA009120(R.M.P), and M01-RR00865(UCLA  General Clinical Research Center); Henry E. Singleton Brain Cancer Research Fund; George Rathmann Family  Foundation; Sidney Kimmel Cancer Research Foundation; and Jonsson Cancer Center Foundation. \| \| --- \|   **Clinical data source:**  **Tumor characteristics WHO grade:** New or recurrent IV  **Previous treatment:** CT/ICH  **DC Arm Injection:** DCs loaded with ATP  **Culture of DC cells:** GM-CSF, IL-4  The data were extracted from Material and methods of Liau LM’s paper.  **Clinical trial phase:** Ⅰ  The data were extracted from the Abstract of Liau LM’s paper.  **Patients (male) and control:** 12(5)/99(UK)  **Median age:** 40.4/＜50  **DC regimens:** 1-10×10^6^/course  **Pre-Therapy KPS:** ≥60  The above data were extracted from Table 1 of Liau LM’s paper.  **OS%(Control): PFS%(Control):**  **0.5 year:** 100(95) **0.5 year:** 83(69)  **1 year:** 100(78) **1 year:** 52(33)  **1.5 year:** 76(39) **1.5 year:** 52(16)  **2 year:** 58(19) **2 year:** 38(4)  **3 year:** 50(7) **3 year:** 14(1.2)  **4 year:** 18(3) **4 year:** 14(1.2)  **Above 5 years:** 29(0) **Above 5 year:** -  The above data were extracted from Figure 1 of Liau LM’s paper. | Number of patients  (control） | OS%(Control) |
| Liau LM et al. 2011 was listed in our papers as reference 20. |  |  |

| **9 clinical trials for meta-analysis** |  |  |
| --- | --- | --- |
| 5. Kikuchi T, Akasaki Y, Irie M, Homma S, Abe T, Ohno T. Results of a phase I clinical trial of vaccination of glioma patients with fusions of dendritic and glioma cells. Cancer Immunol Immunother. 2001 Sep;50(7):337-44. PMID:11676393.  **Participants：**Glioma patients  **Interventions：**Dendritic cell vaccination  **Comparison：**By comparing the immunotherapy group and no-immunotherapy group to investigate the safety and clinical responses of dendritic cell therapy for patients  **Outcomes：**Survival and adverse events  **Setting：**Before and after self control study   \| **Funding sources:** No disclosure. \| \| --- \|   **Clinical data source:**  **Tumor characteristics WHO grade:** New or recurrent unknown  **Clinical trial phase:** Ⅰ  The data were extracted from the title of Kikuchi T’s paper.  **Patients (male) and control:** 8(7)  **Median age:** 38  **Pre-Therapy KPS:** Median 70  The above data were extracted from the table 1 of Kikuchi T’s paper.  **Previous treatment:** SR/CT/RT  **DC Arm Injection:** DCs fused with AIT (i.d)  **Culture of DC cells:** GM-CSF, IL-4,TNF-α  The above data were extracted from Material and methods of Kikuchi T’s paper.  **DC regimens:** 2.4-8.7×10^6^/course  The data were extracted from Table 2 of Kikuchi T’s paper.  **Immunity response:**  **Pre-DC treatment Post-DC treatment**  **CD3+CD8+**: 35.6±8.8; 43.9±5.2;  **CD3+CD4+**: 37±5.2; 31.4±5.31;  **CD56+**: 26.072±12.8; 30.088±13.1;  **CD16+**: 16.8±15; 18.9±9.48;  **IFNγ**  Target cell Autologous giloma cells(P):  **Pre:**45.475±3.725 **Post:**97.85±8.525  The above data were extracted from Table 3 and 4 of Kikuchi T’s paper. | Number of patients  (control） | OS%(Control) |
| Kikuchi T et al. 2001 was listed in our papers as reference 21. |  |  |

| **9 clinical trials for meta-analysis** |  |  |
| --- | --- | --- |
| 6. Yamanaka R, Abe T, Yajima N, Tsuchiya N, Homma J, Kobayashi T, Narita M, Takahashi M, Tanaka R. Vaccination of recurrent glioma patients with tumour lysate-pulsed dendritic cells elicits immune responses: results of a clinical phase I/II trial. Br J Cancer. 2003 Oct 6;89(7):1172-9. PMID: 14520441.  **Participants：**Recurrent glioma patients  **Interventions：**Tumour lysate-pulsed dendritic cells  **Comparison：**This study demonstrated the safety and antitumour effects of autologous tumour lysate-pulsed dendritic cell therapy for patients by comparing the immunotherapy group and no-immunotherapy group.  **Outcomes：**Survival and adverse events  **Setting：**Before and after self control study   \| **Funding sources:** No disclosure. \| \| --- \|   **Clinical data source:**  **Tumor characteristics WHO grade:** New or recurrent unknown  **Clinical trial phase:** I/II  The data were extracted from the title of Yamanaka R’s paper.  **Patients (male) and control:** 10(4)  **Median age:** 46  **Pre-Therapy KPS:** Median 54  The above data were extracted from the table 1 of Yamanaka R’s paper.  **Previous treatment:** SR/RT  **DC Arm Injection:** DCs loaded with ATL (i.d)  **Culture of DC cells:** GM-CSF, IL-4, KLH  The above data were extracted from Material and methods of Yamanaka R’s paper.  **DC regimens:** 10-137.2×10^6^/course  The data were extracted from Table 2 of Yamanaka R’s paper.  **Immunity response:**  **Pre-DC treatment**  **CD3+CD8+**: 35.6±8.8;  **CD3+CD4+**: 37±5.2;  **CD56+**: 26.072±12.8;  **CD16+**: 16.8±15;  The data were extracted from the Table 3 of Yamanaka et al.'s paper | Number of patients  (control） | OS%(Control) |
| Yamanaka R et al. 2003 was listed in our papers as reference 22. |  |  |

| **9 clinical trials for meta-analysis** |  |  |
| --- | --- | --- |
| 7. Yu JS, Liu G, Ying H, Yong WH, Black KL, Wheeler CJ. Vaccination with tumor lysate-pulsed dendritic cells elicits antigen-specific, cytotoxic T-cells in patients with malignant glioma. Cancer Res. 2004 Jul 15;64(14):4973-9. PMID: 15256471.  **Participants：**Malignant glioma patients  **Interventions：**Tumor lysate-pulsed dendritic cells  **Comparison：**By comparing the immunotherapy group and no-immunotherapy group to assess the safety and bioactivity of tumor lysate-pulsed dendritic cell (DC) vaccination to treat patients.  **Outcomes：**Survival  **Setting：**Historical control   \| **Funding sources:** This work was funded in part by NIH grant NS02232-01 to J. S. Yu. \| \| --- \|   **Clinical data source:**  **Tumor characteristics WHO grade:** New or recurrent unknown  **Clinical trial phase:** Ⅰ  The data were extracted from the abstract of Yu JS’s paper.  **Patients (male) and control:** 14(10)/26(UK)  **Median age:** 45/53  **Pre-Therapy KPS:** ≥60  The above data were extracted from the table 1 of Yu JS’s paper.  **Previous treatment:** SR/CT  **DC Arm Injection:** DCs loaded with ATL (i.d)  **Culture of DC cells:** GM-CSF, IL-4  **DC regimens:** 10^7^-10^8^/course  The above data were extracted from Material and methods of Yu JS’s paper.  **OS%(Control):**  **0.5 year:** 100(55)  **1 year:** 85(30)  **1.5 year:** 85(10)  **2 year:** 60(10)  **3 year:** 35(10)  **4 year:** 35(0)  **Above 5 years:** -  The above data were extracted from Figure 4 of Yu JS’s paper. | Number of patients  (control） | OS%(Control) |
| Yu JS et al. 2004 was listed in our papers as reference 23. |  |  |

| **9 clinical trials for meta-analysis** |  |  |
| --- | --- | --- |
| 8. Jie X, Hua L, Jiang W, Feng F, Feng G, Hua Z. Clinical application of a dendritic cell vaccine raised against heat-shocked glioblastoma. Cell Biochem Biophys. 2012 Jan;62(1):91-9. doi: 10.1007/s12013-011-9265-6. PMID:21909820.  **Participants：**Glioblastoma patients  **Interventions：**Dendritic cell vaccine  **Comparison：**By comparing the immunotherapy group and no-immunotherapy group to determine the efficacy of the vaccine in a clinical trial.  **Outcomes：**Survival  **Setting：**Randomized controlled trial   \| **Funding sources:** No disclosure. \| \| --- \|   **Clinical data source:**  **Tumor characteristics WHO grade:** Recurrent IV  **Clinical trial phase:** Ⅰ/Ⅱ  **Previous treatment:** SR/CT/RT  **DC Arm Injection:** DCs loaded with AHL and GM-CSF(s.c)  **Culture of DC cells:** GM-CSF, IL-4, IL-1β, PGE2, TNF-α  **DC regimens:** 6×10^6^/course  The above data were extracted from Material and methods of Jie X’s paper.  **Patients (male) and control:** 13(10)/12(9)  **Median age:** 40.2/43.1  The above data were extracted from the table 2 of Jie X’s paper.  **Pre-Therapy KPS:** ≥60  The above data were extracted from Table 1 of Jie X’s paper.  **OS%(Control): Immunity response:**  **0.5 year:** 93(100) **Pre-DC treatment Post-DC treatment**  **1 year:** 72(41) **CD3+CD8+**: 35.6±8.8; 43.9±5.2;  **1.5 year:** 48(19) **CD3+CD4+**: 37±5.2; 31.4±5.31;  **2 year:** 0(0) **CD56+**: 26.072±12.8; 30.088±13.1;  **3 year:** 0(0) **CD16+**: 16.8±15; 18.9±9.48;  **4 year:** 0(0) **IFNγ**  **Above 5 years:** - Pre:22.23±6.46 Post:76.23±16.18  The above data were extracted from Figure 1 and Table 3 and 4 of Jie X’s paper. | Number of patients  (control） | OS%(Control) |
| Jie X et al. 2004 was listed in our papers as reference 24. |  |  |

| **9 clinical trials for meta-analysis** |  |  |
| --- | --- | --- |
| 9. Cho DY, Yang WK, Lee HC, Hsu DM, Lin HL, Lin SZ, Chen CC, Harn HJ, Liu CL, Lee WY, Ho LH. Adjuvant immunotherapy with whole-cell lysate dendritic cells vaccine for glioblastoma multiforme: a phase II clinical trial. World Neurosurg. 2012 May-Jun;77(5-6):736-44. doi: 10.1016/j.wneu.2011.08.020. Epub 2011 Nov 7.PMID: 22120301.  **Participants：**Glioblastoma multiforme patients  **Interventions：**Whole-cell lysate dendritic cells vaccine  **Comparison：**By comparing the immunotherapy group and no-immunotherapy group to evaluate effectiveness of autologous dendritic cell vaccine for glioblastoma multiforme.  **Outcomes：**Survival, KPS, PFS  **Setting：**Randomized controlled trial   \| **Funding sources:** No disclosure. \| \| --- \|   **Clinical data source:**  **Tumor characteristics WHO grade:** New IV  **Previous treatment:** SR/CT/RT  **DC Arm Injection:** DCs loaded with ATL (s.c)  **DC regimens:** 2-5×10^7^/course  **Culture of DC cells:** GM-CSF, IL-4  The above data were extracted from Material and methods of Cho DY’s paper.  **Clinical trial phase:** Ⅱ  The data were extracted from title of Cho DY’s paper.  **Patients (male) and control:** 18(8)/16(8)  **Median age:** 58.6/55.8  The above data were extracted from the table 2 of Cho DY’s paper.  **Pre-Therapy KPS:** >70  The above data were extracted from Table 1 of Cho DY’s paper.  **OS%(Control): PFS%(Control):**  **0.5 year:** - **0.5 year:** 69(63)  **1 year:** 88.9(75) **1 year:** 52(33)  **1.5 year:** - **1.5 year:** 52(9)  **2 year:** 44.4(18.8) **2 year:** 33(0)  **3 year:** 16.7(0) **3 year:** 33(0)  **4 year:** - **4 year:** 33(0)  **Above 5 years:** - **Above 5 year:** -  **KPS:**  **Pre-DC treatment Post-DC treatment**  85.6±8 36.1±37.7  The above data were extracted from Table 1 and Figure 4 of Cho DY’s paper. | Number of patients  (control） | OS%(Control) |
| Cho DY et al. 2012 was listed in our papers as reference 25. |  |  |
